# Assembly of Differently Sized Supercharged Protein Nanocages into Superlattices for Construction of Binary Nanoparticle–Protein Materials

**DOI:** 10.1021/acsnano.4c09551

**Published:** 2024-08-27

**Authors:** Michael Rütten, Laurin Lang, Henrike Wagler, Marcel Lach, Niklas Mucke, Ulrike Laugks, Carolin Seuring, Thomas F. Keller, Andreas Stierle, Helen M. Ginn, Tobias Beck

**Affiliations:** † Institute of Physical Chemistry, Department of Chemistry, University of Hamburg, Hamburg 20146, Germany; ‡ The Hamburg Centre for Ultrafast Imaging, University of Hamburg, Hamburg 20146, Germany; § Centre for Structureal Systems Biology (CSSB), Hamburg 22607, Germany; ∥ Department of Structural Cell Biology of Viruses, Leibniz Institute of Virology, Hamburg 20251, Germany; ⊥ Department of Chemistry, University of Hamburg, Hamburg 20146, Germany; # Centre for X-ray and Nano Science (CXNS), Deutsches Elektronen-Synchrotron DESY, Hamburg 22607, Germany; ∇ Department of Physics, University of Hamburg, Hamburg 22607, Germany; ○ Center for Free-Electron Laser Science (CFEL), Deutsches Elektronen-Synchrotron DESY, Hamburg 22607, Germany; ◆ Institute for Nanostructure and Solid State Physics, Department of Physics, University of Hamburg, Hamburg 22761, Germany

**Keywords:** biohybrid nanomaterials, binary superlattices, protein cages, inorganic nanoparticles, SAXS, single-crystal small-angle X-ray diffraction SC-SAXD, electrostatic assembly

## Abstract

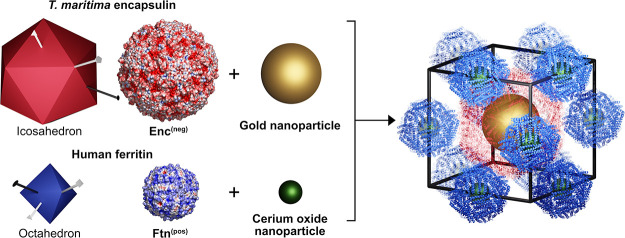

This study focuses on the design and characterization of binary nanoparticle superlattices: Two differently sized, supercharged protein nanocages are used to create a matrix for nanoparticle arrangement. We have previously established the assembly of protein nanocages of the same size. Here, we present another approach for multicomponent biohybrid material synthesis by successfully assembling two differently sized supercharged protein nanocages with different symmetries. Typically, the ordered assembly of objects with nonmatching symmetry is challenging, but our electrostatic-based approach overcomes the symmetry mismatch by exploiting electrostatic interactions between oppositely charged cages. Moreover, our study showcases the use of nanoparticles as a contrast enhancer in an elegant way to gain insights into the structural details of crystalline biohybrid materials. The assembled materials were characterized with various methods, including transmission electron microscopy (TEM) and single-crystal small-angle X-ray diffraction (SC-SAXD). We employed cryo-plasma-focused ion beam milling (cryo-PFIB) to prepare lamellae for the investigation of nanoparticle sublattices via electron cryo-tomography. Importantly, we refined superlattice structure data obtained from single-crystal SAXD experiments, providing conclusive evidence of the final assembly type. Our findings highlight the versatility of protein nanocages for creating distinctive types of binary superlattices. Because the nanoparticles do not influence the type of assembly, protein cage matrices can combine various nanoparticles in the solid state. This study not only contributes to the expanding repertoire of nanoparticle assembly methods but also demonstrates the power of advanced characterization techniques in elucidating the structural intricacies of these biohybrid materials.

In materials science, it is of paramount importance to fabricate materials with well-defined composition and intricate structures, because those features directly affect the functional properties of the synthesized materials. A detailed understanding of the impact of the material composition and structural design enables tailoring the materials’ properties to specific applications.^1−3^ Along these lines, combining more than one type of building block into binary materials can generate properties that go beyond those of individual components, with interactions between the building blocks giving rise to emerging properties.^4,5^ With atoms as building blocks, various binary structural motifs are known for inorganic compounds, such as simple AB or AB_2_,^6,7^ but also more sophisticated structural types such as perovskite or spinel-type.^7−9^ Generally, the two building blocks for these inorganic materials, usually two ions with opposing charges (anion and cation), can have different sizes as well.^10,11^

By assembling nanoparticles, researchers have on the one hand tried to mimic the large structural variety of these inorganic assembly types but also strived to go beyond these motifs. Because nanoparticles have a completely different set of properties, such as magnetic,^12^ catalytic,^13^ or optical properties,^14^ they enable the construction of superlattices with properties surpassing those of atomic or molecular lattices.^15−17^ The assembly of nanoparticles into materials can be achieved, for example, using the interaction of the nanoparticle ligand shell that stabilizes the particles in colloidal suspensions.^18,19^ Other sophisticated methods include DNA as mediating linkers, which can specifically assemble particles.^20^ Some of these nanoparticle assemblies are analogous to inorganic structures, now with nanoparticles as the building blocks. Interestingly, several nanoparticle superlattices synthesized in this way show previously unknown assembly types, with no direct analogue found in inorganic structures.^21−23^ As these approaches use the nanoparticles as the fundamental building block, the structural homogeneity of the particles is crucial for lattice order and domain size. Because improved synthetic procedures can now yield uniform nanoparticles, superlattices with domain sizes from micrometers to millimeters can be achieved.^24−26^ Another approach to highly ordered lattices is to use an atomically defined nanocluster for material assembly.^27^

Protein nanocages offer an alternative route toward highly ordered nanoparticle superlattices using an atomically defined template.^28−30^ For unitary lattices, one type of protein cage is assembled into 3D structures.^31^ For binary assemblies, two oppositely charged protein nanocages can be combined to yield two-component structures, enabling the formation of binary nanoparticle lattices.^32^ The protein nanocage-based assembly shows several advantages: Due to their precisely defined shape and, thus, inherent high monodispersity, superlattices with a high degree of order and large domain sizes can be obtained.^32,33^ Along these lines, because the protein nanocages retain their defined sizes after cargo encapsulation, the cages overwrite any size dispersity of cargo particles. Moreover, the type of assembly and the high degree of order only depend on the protein cages but not on the type of cargo inside the cages.^32^ Therefore, only a part of the cages can be filled with nanoparticles and combined with empty cages to finely tune the optical response. We have recently demonstrated this placeholder feature to construct nanomaterials based on protein cages and plasmonic gold nanoparticles. These metacrystals show anomalous refraction of visible light.^34^ Moreover, with the protein cages, two types of cargoes can be assembled into 3D lattices. We have used binary lattices based on the ferritin cage to combine plasmonic gold nanoparticles and fluorescent dyes in the solid state and studied the energy transfer between these cargoes using fluorescence lifetime imaging.^35^

However, the advantage of the protein cage’s uniform size also poses a challenge, because modifying the cage size to control the lattice parameters is not as straightforward as, for example, extending the length of a DNA linker in DNA-based nanoparticle assembly.^36,37^ Because it is not easily possible to extend the size of one protein cage, smaller and larger protein cages need to be selected, i.e., different protein cage types. Moreover, larger protein cages are particularly interesting as the increased size of the cavity grants more space for cargo molecules or nanoparticles.^38,39^ In the current study, we used two differently sized protein cages, supercharged on the surface to yield cages with opposing charges. Because these are two different types of protein nanocages, these cages also have different types of symmetry, i.e., the arrangement of the protein subunits forming the protein cage.^40,41^ Importantly, the assembly of these differently sized protein cages presents a challenge for the construction of biohybrid materials in the crystalline state, because the differing symmetries complicate repeating interactions between the cages. We envisioned that assembly based on supercharged protein cages can overcome these symmetry constraints because the charge-driven assembly does not require defined interaction sites but is rather based on the electrostatic interactions between the oppositely charged cages.

## Results and Discussion

**Figure 1:**
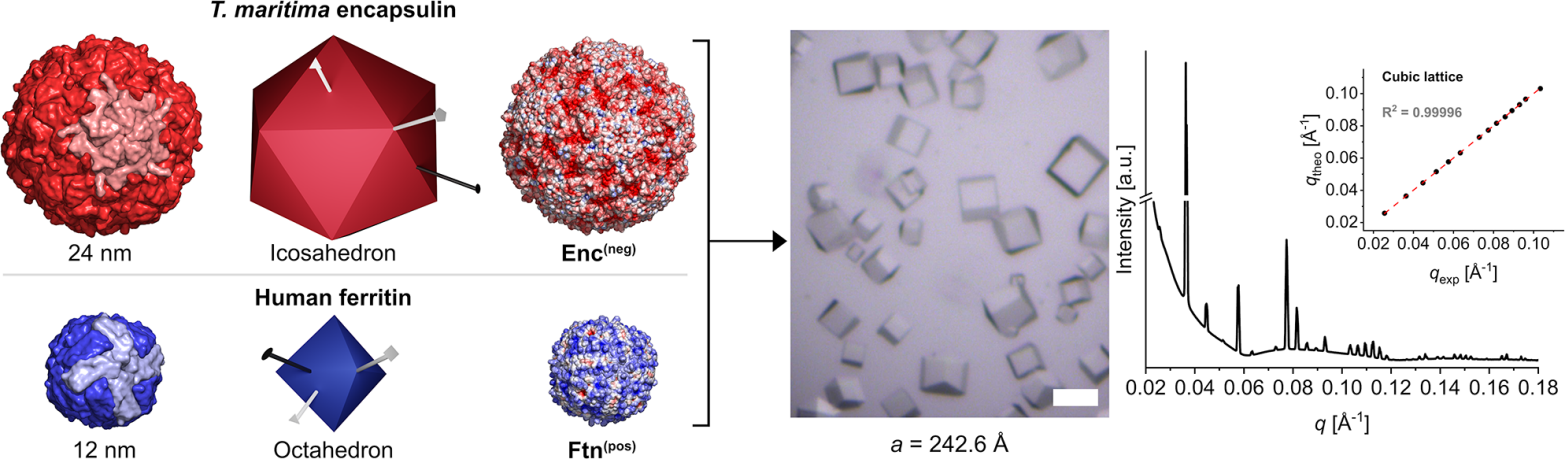
Structural and physical differences between encapsulin and ferritin protein cages and their crystalline assembly investigated via small-angle X-ray scattering. Left panels: The *T. maritima* encapsulin container has an outer diameter of 24 nm, icosahedral symmetry, and a negative surface charge, Enc^(neg)^. On the other hand, human ferritin has an outer diameter of 12 nm and octahedral symmetry. Moreover, the ferritin variant used here features a positive surface charge, Ftn^(pos)^. The surface charge is coded from red to blue from −5 to 5 kT/e. The assembly of the two cages forms the crystalline sample, composed of several crystals (optical microscopy image, scale bar equals 100 μm, unit cell given below the image), and it was investigated via SAXS (right panel). The correlation between experimental and theoretical *q* values is shown in the inset.

To realize the assembly of binary superlattices based on two differently sized protein cages, we chose the two protein cages encapsulin and ferritin. These differ in size (24 nm vs 12 nm outer diameter, Figure 1) and symmetry (icosahedral vs octahedral, Figure 1). For the assembly, we created a variant, namely, the negatively charged encapsulin Enc^(neg)^ based on the wild-type *Thermotoga maritima* encapsulin (*T. maritima* encapsulin, Figures S1–S3). This encapsulin variant contains an additional mutation that removes a side chain responsible for flavin binding (W90E) (Table S1).^42,43^ The elimination of the flavin binding site is important for the assembly process because interactions should exclusively involve electrostatic or protein–protein interactions while excluding any additional interactions based on the flavin moiety. The second protein nanocage is human ferritin. Here, we selected the variant Ftn^(pos)^, which was positively supercharged with the help of computational protein design in earlier work.^44^ Ferritin has a size of 12 nm, making it half the size of the encapsulin nanocage. Therefore, these two cages are ideally suited to construct binary lattices as the encapsulin cage has a large cavity. Because these cages have different sizes, assembly would likely yield a structure more intricate than a simple AB assembly. We expected that the two cages could be assembled based on the complementary charge of the two cages, because zeta potential measurements show for encapsulin variant Enc^(neg)^ ζ = −24.5 mV and for the ferritin variant Ftn^(pos)^ ζ = +19.2 mV. Using a standard protein crystallization setup (for details, please refer to the Supporting Information), we identified an assembly condition that yielded cubic crystals with a dimension of around 100 μm. We aimed to determine the structure of the protein matrix using single-crystal protein crystallography. Yet, the resolution was insufficient to determine the molecular structure. However, despite the low resolution of the collected data, the crystal system and unit cell parameters were determined. A cubic lattice with *a* = 242.6 Å was determined. To obtain more details on the lattice type, small-angle X-ray scattering (SAXS) of an ensemble of protein crystals was carried out to confirm the cubic lattice. The theoretical *q* values fit very well to the experimentally determined *q* values (Figure 1; for details on how theoretical *q* values were obtained, see the Supporting Information).

Due to the low resolution of the acquired crystallographic data, we could not determine the molecular structure of the protein matrix. However, first indications from the crystallization setups showed that both protein cages are likely present in the obtained crystals because both cages are needed for crystal formation: The same assembly conditions as for binary assembly but with only one of either cage in the crystallization drop did not yield any crystal formation. Next, we confirmed that both cages are contained within the crystals using experiments based on size-exclusion chromatography and gel electrophoresis. With both methods, it is possible to discriminate between the two nanocages encapsulin and ferritin, exploiting the significant difference of 1.3 MDa in their molecular masses (Enc^(neg)^ 1.8 MDa and Ftn^(pos)^ 0.5 MDa). First, both protein cages were cocrystallized in batch. The supernatant from this crystallization batch was analyzed with Fast Protein Liquid Chromatography (FPLC, Figures S4–S7 and Tables S5–S7). Moreover, a solution of the dissolved crystals was characterized with sodium dodecyl sulfate polyacrylamide gel electrophoresis (SDS-PAGE, Figures S8 and S9 and Tables S8 and S9). The outcome of both techniques gave information regarding the protein composition, namely, a higher ratio of Ftn^(pos)^ relative to Enc^(neg)^. Strikingly, a mean ratio of about 3 Ftn^(pos)^ nanocages to 1 Enc^(neg)^ nanocage was determined (FPLC: 3.5 to 1; SDS-PAGE: 3.3 to 1, Table S10). Although this information only gives an approximate ratio of Ftn^(pos)^ to Enc^(neg)^ within the unit cell, it can also serve as a starting point for further structural analysis despite lacking the positions and orientation of the nanocages.

To obtain more details on the structural composition, the idea emerged to utilize nanoparticle-loaded protein crystals for further analysis. Employing gold or cerium oxide nanoparticles (NPs), which have a high electron density compared to protein nanocages (mainly carbon-based), enabled enhanced visualization, especially in electron microscopy settings. Unlike proteins, these nanoparticles provide a strong contrast without necessary staining. Moreover, due to their high electron density, nanoparticle-containing hybrids should also show increased X-ray scattering and diffraction.

**Figure 2:**
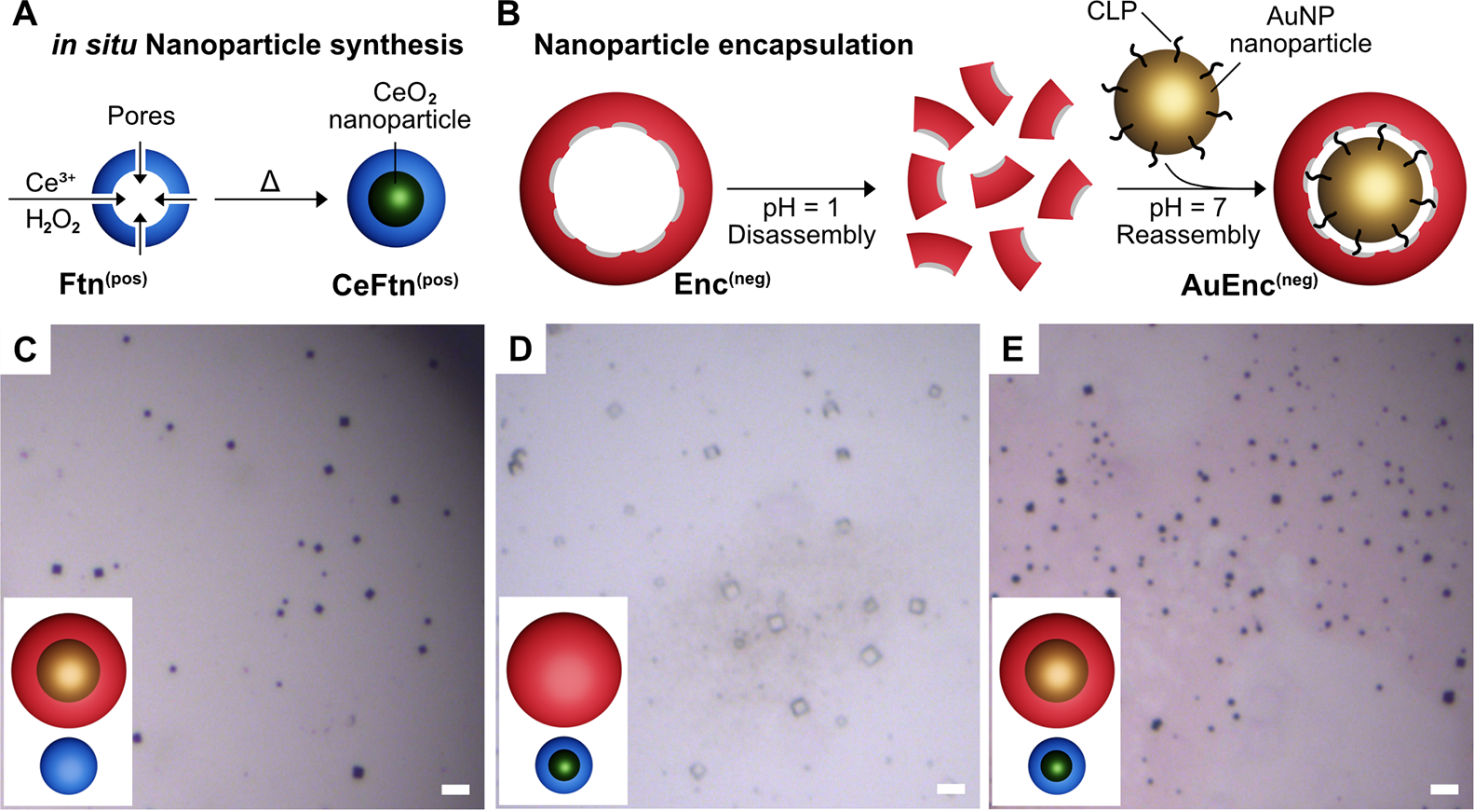
Overview of the nanoparticle-loading procedure and crystals based on nanoparticle-loaded protein cages. (A) The *in situ* synthesis of CeO_2_ nanoparticles inside the ferritin cavity is shown. Precursors diffuse inside the cavity through the pores of the nanocage. Particles are synthesized at higher temperature. (B) For the second cage, gold nanoparticles are encapsulated into the encapsulin container by dis- and reassembly. During reassembly, the *ex situ*-synthesized nanoparticles are added and encapsulated by a peptide-directed encapsulation process. (C–E) Protein cages can be crystallized in different combinations: eFtn^(pos)^ with AuEnc^(neg)^, CeFtn^(pos)^ with eEnc^(neg)^, and CeFtn^(pos)^ with AuEnc^(neg)^. The scale bars equal 50 μm.

The loading of cerium oxide (CeO_2_) NPs into Ftn^(pos)^ (referred to as CeFtn^(pos)^) was carried out according to an already reported protocol.^32^ Notably, the synthesis of NPs within the cavity does not require the need for dis- and reassembly. The CeO_2_ particles are synthesized *in situ* based on the diffusion of precursor ions and molecules into the ferritin cavity, where particle formation occurs (Figure 2A and Figure S10). After NP synthesis, the NP-loaded CeFtn^(pos)^ nanocage was subjected to size-exclusion chromatography (SEC) for purification (Figure S11) to yield pure samples suitable for assembly. Next, Enc^(neg)^ was loaded with gold nanoparticles (AuNPs). According to a previously established protocol with slight modifications, encapsulation of 13 nm AuNPs (Figure S12) within Enc^(neg)^ was achieved through a cargo-loading peptide (CLP)-mediated approach.^38^ This strategy makes use of specific interactions between CLP-binding pockets on the interior of the Enc^(neg)^ cavity and the CLP-functionalized NPs, causing highly efficient encapsulation. The encapsulation process begins with the disassembly of the nanocage followed by its reassembly in the presence of NPs (Figure 2B). After encapsulation, the AuNP-loaded Enc^(neg)^ (AuEnc^(neg)^) was purified via ion-exchange chromatography (IEC) to remove potential free NPs and SEC in preparation for crystallization (Figures S13 and S14). With both protein nanocages loaded with different types of NPs, the capability to generate heterobinary crystals composed of two different types of materials was tested. We tried different combinations, either having only one cage filled and the other one empty or AuEnc^(neg)^ and CeFtn^(pos)^ both filled with nanoparticles (Figure 2C–E). Crystals comprising AuEnc^(neg)^ and empty Ftn^(pos)^ (eFtn^(pos)^) appeared as black crystals, exhibiting a pronounced decrease in size to approximately 20 μm (Figure 2C). The observed change in color can be attributed to the presence of encapsulated AuNPs. In contrast, crystals containing empty Enc^(neg)^ (eEnc^(neg)^) and CeFtn^(pos)^ exhibited a transparency, reaching dimensions of up to 40 μm (Figure 2D). Furthermore, the combination of both filled cages, AuEnc^(neg)^ and CeFtn^(pos)^, also yielded dark crystals of similar dimensions of around 20 μm (Figure 2E).

Interestingly, as noted above, the nanoparticle-loaded crystals show a decrease in size compared to crystals of empty cages. We attribute this to the fact that the dispersity of the loaded cages increases slightly, as shown in dynamic light scattering measurements (Figure S15). The loading procedure increases the polydispersity index (PDI) for both building blocks.

With these nanoparticle-filled crystals, we used a range of methods to further characterize the binary assembly. We first turned to electron microscopy to utilize the enhanced contrast of the nanoparticles. Importantly, by loading only one nanocage with nanoparticles, but still having the other empty cage present in the lattice, we envisioned that we could determine the details of each sublattice first and later combine this information. In detail, scanning electron microscopy (SEM) and transmission electron microscopy (TEM) were applied. With these measurements, we expected to characterize the nanoparticle lattices.

**Figure 3:**
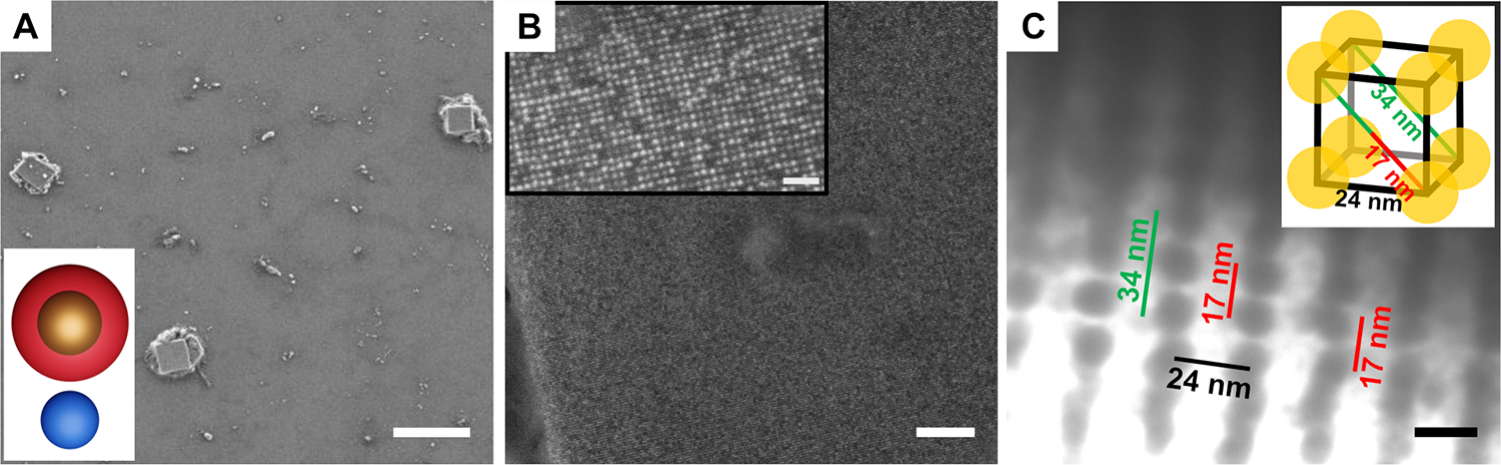
Electron microscopy analysis of AuNP-loaded Enc^(neg)^ and empty Ftn^(pos)^ crystals. (A) Heterobinary crystals based on AuEnc^(neg)^/eFtn^(pos)^ were transferred on Si-wafer and investigated via scanning electron microscopy. Scale bar: 15 μm. (B) Further zoom on the crystal surface reveals a cubic arrangement of spheres (see the inset). Scale bars: 500 nm and 100 nm (inset). (C) Transmission electron microscopy enabled the visualization of a well-ordered nanoparticle lattice. Measured distances fit to the (101) plane of a cubic crystal system (see the inset). Scale bar: 10 nm.

We first looked into the encapsulin sublattice. SEM measurements were carried out with a heterobinary AuEnc^(neg)^/eFtn^(pos)^ crystal on a Si-wafer (Figure 3A). To visually verify the cubic arrangement of the protein nanocages, one crystal was examined more closely. In detail, spherical objects of one size were observed (Figure 3B). The spherical objects were 24 nm large, indicating that only AuEnc^(neg)^ was visualized. Due to the AuNP loading, the Enc^(neg)^ nanocage gave stronger contrast than eFtn^(pos)^. The cubic lattice of the crystal could clearly be observed as bright objects were on the top layer (no contamination of the surface). Nanocages of lower levels appeared grayish due to having less intensity. The surface featured darker spots, which are holes indicating a missing nanocage in one layer. As the objects are closely packed, the distances between the nanocages were around 24 nm and fit very well to the previously determined unit cell parameter of 24.26 nm. In atomic force microscopy (AFM)^45^ measurements, the AuNP-loaded protein nanocages were visible in phase imaging and roughly 24 nm apart from each other (Figure S16). After milling the crystal with a focused ion beam (FIB), the cubic arrangement could be observed in SEM as well scanning transmission electron microscopy (STEM, Figures S17 and S18).^45^ We could not image the smaller eFtn^(pos)^ in any of the three methods, indicating that through the nanoparticle encapsulation, the AuNP-loaded Enc^(neg)^ nanocage seems to get more rigid, causing a stronger contrast and being well pronounced in both SEM and AFM imaging. Moreover, a heterobinary AuEnc^(neg)^/eFtn^(pos)^ crystal was analyzed with TEM. Due to the low electron density, the protein nanocage is not visible in TEM without further staining and only AuNPs encapsulated in Enc^(neg)^ are visible (Figure 3C). The cubic crystal is a few micrometers thick, therefore too thick to be imaged in TEM. Nevertheless, thinner parts of the crystal seemed suitable for imaging the crystal in one direction. Well-ordered AuNPs of a periodic lattice are observed. Distances between AuNPs can be attributed to the (101) plane of a cubic crystal system (Figure 3C, inset). The determined distances of the AuNPs in TEM further strengthen and verify the presence of a cubic crystal system.

We then turned to the ferritin sublattice. Interestingly, crystals composed of eEnc^(neg)^/CeFtn^(pos)^ could not be characterized by AFM and SEM using the same parameters as shown above (Figures S19–S21). Apparently, due to the smaller sizes, the CeO_2_ NPs do not yield such a high contrast as the AuNPs (Figure 3 and Figure S17). Moreover, eEnc^(neg)^/CeFtn^(pos)^ crystals are too thick for TEM imaging. Therefore, to image the 3D lattice, thin lamellae were prepared at cryonic temperatures to enable higher resolution.

**Figure 4:**
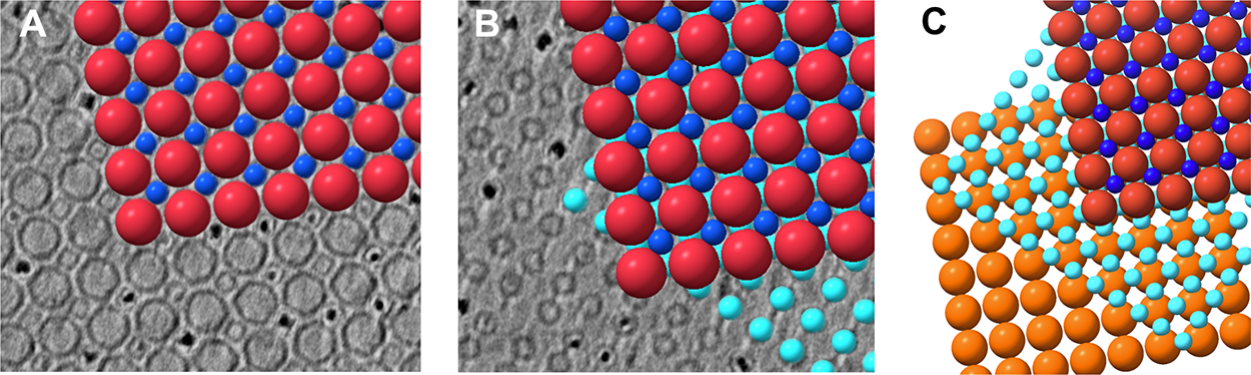
Electron cryo-tomogram of a lamella from an eEnc^(neg)^/CeFtn^(pos)^ crystal. (A) One plane of the tomogram, with encapsulin eEnc^(neg)^ forming a cubic lattice (red spheres with a diameter of 24 nm) and ferritin cages CeFtn^(pos)^ (dark blue spheres with a diameter of 12 nm). (B) Plane parallel to the plane in panel (A) but at a different *z* level. Ferritin cages CeFtn^(pos)^ (light blue spheres with a diameter of 12 nm). (C) Model of the sublattices shown in panel (B), with another plane eEnc^(neg)^ added (orange spheres with a diameter of 24 nm).

Lamellae suitable for high-resolution cryo-tomography were prepared from eEnc^(neg)^/CeFtn^(pos)^ protein crystals by focused ion beam milling and imaged by electron cryo-transmission microscopy.^46^ The micrographs (Figure S22) showed a cubic arrangement of the CeO_2_ NPs, in agreement with the fast Fourier transformation (FFT, Figure S22B). To corroborate the observations, electron cryo-tomography data acquisition was employed to image the superlattices at higher complexity and in 3D. For these experiments, crystals composed of eEnc^(neg)^/CeFtn^(pos)^ were selected, which had a lower loading of CeO_2_ NPs. This allowed the investigation of differences between loaded and empty ferritin cages within the crystal lattice. This time, we employed cryo-plasma-focused ion beam milling–scanning electron microscopy (cryo-PFIB-SEM)^47^ for lamella preparation. Grids containing lamellae were then transferred in high-resolution cryo-TEM to acquire electron cryo-tomography data employing a dose-symmetric tilt scheme starting at 0° tilt angle and capturing alternating negative and positive tilts in 2° increments. Reconstruction of the data allowed investigation of the structural details of the crystalline sample as depicted in Figure 4. Within these thin lamellae, the protein nanocages and CeO_2_ NPs were visualized, and they indicate a cubic arrangement. Figure S23 shows the raw data.

For the analysis, the tomogram was rotated (see details in Experimental Methods) so that the *z* axis is parallel to one plane of eEnc^(neg)^ cages (Figure 4A). The eEnc^(neg)^ cages form a highly ordered cubic lattice (red spheres) with CeFtn^(pos)^ (dark blue spheres) located in between. A plane parallel to the plane in Figure 4A, but at a different *z* level, shows the location of the remaining ferritin cages (light blue), which are located between two eEnc^(neg)^ of the upper plane (Figure 4B). Another plane of encapsulin cages (orange) is shown in Figure 4C. The ferritin cages (dark blue) within the encapsulin plane are located between eEnc^(neg)^ within one plane (coordination number 4), whereas the ferritin cages (light blue) are located between two planes of eEnc^(neg)^ cages (red and orange spheres), also having a coordination number of 4 by making contacts to four neighboring cages. A figure showing the tomogram without the model spheres is found in Figure S23.

In the tomogram, some important structural details of the crystalline assembly could be observed. On the one hand, the location of the ferritin cages clearly indicated a structure type with a stoichiometry of AB_3_ (Figure S24A), because only one ferritin cage between four encapsulin cages was found. A stoichiometry of AB_4_ (Figure S24B) would require additional ferritin cages to be present in the intermediate layer, between the evenly spaced cages (Figure 4B). Moreover, there is no difference between the positioning of ferritin cages filled with CeO_2_ NPs and empty cages in Figure 4A,B. This indicates that the cargo particles did not have any effect on the cage crystal structure. We note that the ordering of the ferritin cages is not as high as the lattice of the encapsulin cages. This could either be an intrinsic property of these crystals or could be due to the preparation process of the crystalline lattice. We have seen that some of the lamellae show deformation, for example, during TEM data collection. Along these lines, we observe, especially at the edges of the lamellae, a decreased high range order, possibly as an artifact of sample preparation.

**Figure 5:**
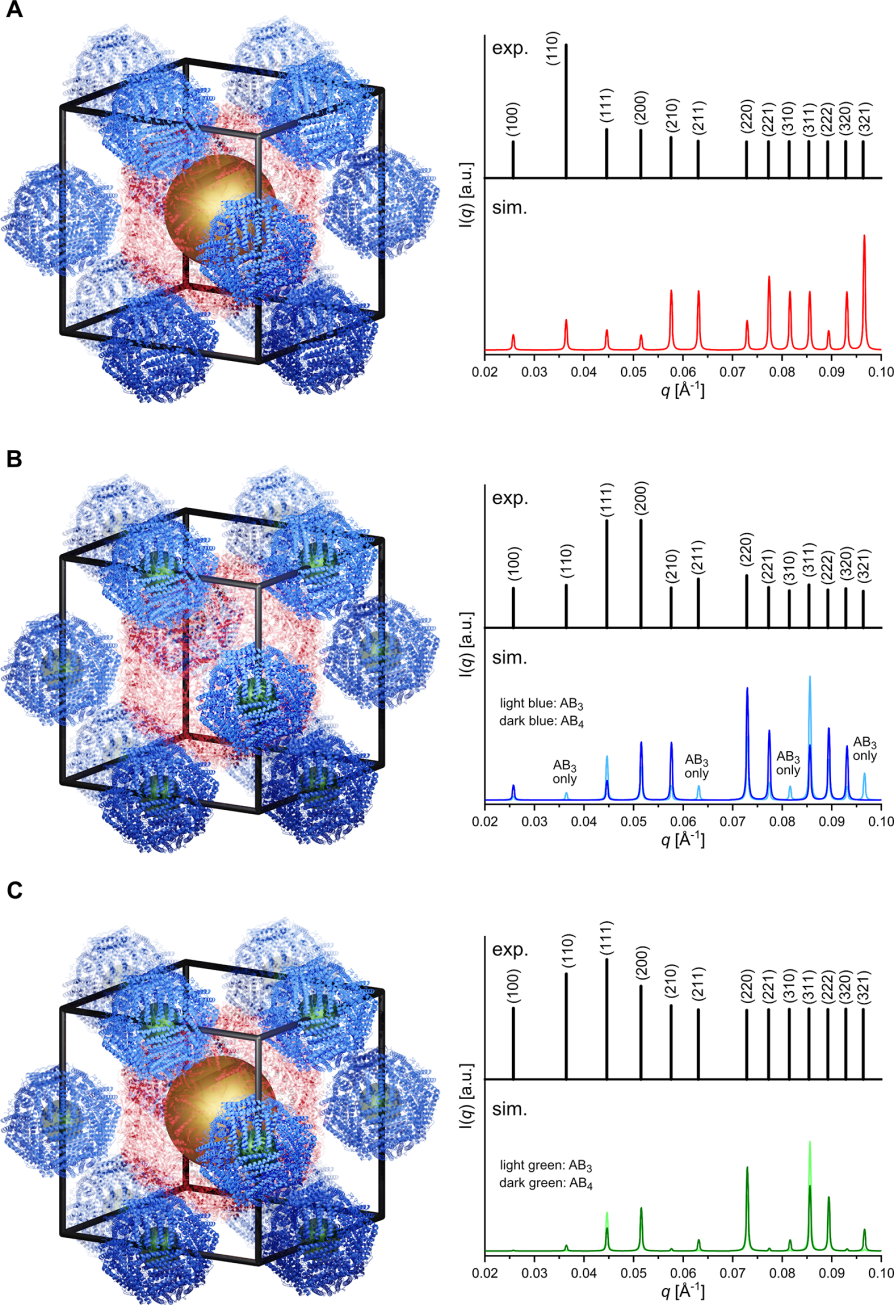
Single-crystal SAXS experiments with heterobinary AuEnc^(neg)^/eFtn^(pos)^ (A), eEnc^(neg)^/CeFtn^(pos)^ (B), and AuEnc^(neg)^/CeFtn^(pos)^ (C) crystals. On the left, models of the unit cell with different NP loadings are shown, while on the right, the experimental data (black) are compared with the simulated data (colored). Simulated data for an AB_3_ structure are indicated by a brighter color, while an AB_4_ structure features a darker color. Reflections only present in structure type AB_3_ are labeled with “AB_3_ only”. (A) The observed reflections for the AuEnc^(neg)^/eFtn^(pos)^ crystal fit to the simulated data (red) of a primitive cubic lattice where only the gold nanoparticle is present within the unit cell. (B) The observed diffraction for the eEnc^(neg)^/CeFtn^(pos)^ crystal fits to an AB_3_ structure with three cerium oxide nanoparticles in one unit cell but does not exclude the presence of unit cells of an AB_4_ structure (blue). (C) For the AuEnc^(neg)^/CeFtn^(pos)^ crystal, the observed reflections fit to the simulated data (green).

To confirm the assumed AB_3_ structure type, we carried out single-crystal X-ray diffraction experiments with the nanoparticle-loaded crystals. Structure determination via diffraction methods might be considered to be indirect, because it does not—in contrast to electron microscopy—visualize the structural arrangement directly but infers the lattice from the diffracted X-ray beam. However, it is a powerful technique because X-ray diffraction characterizes the bulk sample and its 3D structure with high penetration depth, revealing atomic details of not only small molecules but also proteins or the arrangement of nanoparticles, as shown here. Our nanoparticle lattices diffract X-rays in the small-angle regime. Therefore, we refer to the method as single-crystal small-angle X-ray diffraction (SC-SAXD). Data from single-crystal diffraction experiments were integrated and merged (for details, see Experimental Methods). In comparison to the protein lattice of the empty protein crystal (Figure 1), the lattice of different NP-loaded protein crystals showed similar lattice parameters (Table S11). To further confirm the type of assembly as elucidated by electron cryo-tomography, we compared averaged SAXD data and simulated diffraction data. Please note that we did not record a typical SAXS pattern but integrated the diffraction peaks for the SAXD data (example for a diffraction image: Figure S26). First, indexed reflections were averaged and compared with simulated data for a primitive cubic cell (unit cell dimensions are listed in Table S11). All observed reflections for the AuEnc^(neg)^/eFtn^(pos)^ crystal (Figure 5A) fit well to cubic cells with the space group *P*23 or *P*432. To differentiate between AB_3_ and a potential AB_4_ structural type, we looked at systematic absences for the eEnc^(neg)^/CeFtn^(pos)^ crystal (Figure 5B). In detail, for an AB_4_ structure, reflections such as (110), (211), (310), and (321) should be missing due to systematic absences of this assembly type. However, exactly these reflections are present in the experimental data, indicating that the unit cell is indeed at least an AB_3_ structure. For the third sample (Figure 5C), in which both nanocages are loaded, the AuEnc^(neg)^/CeFtn^(pos)^ crystals do not feature characteristic absences for reflections in either the simulation or experimental data. Nevertheless, unit cells simulated with alternative centering do not feature reflections that are observed in these experiments, indicating that both nanocages set up a primitive unit cell.

**Figure 6:**
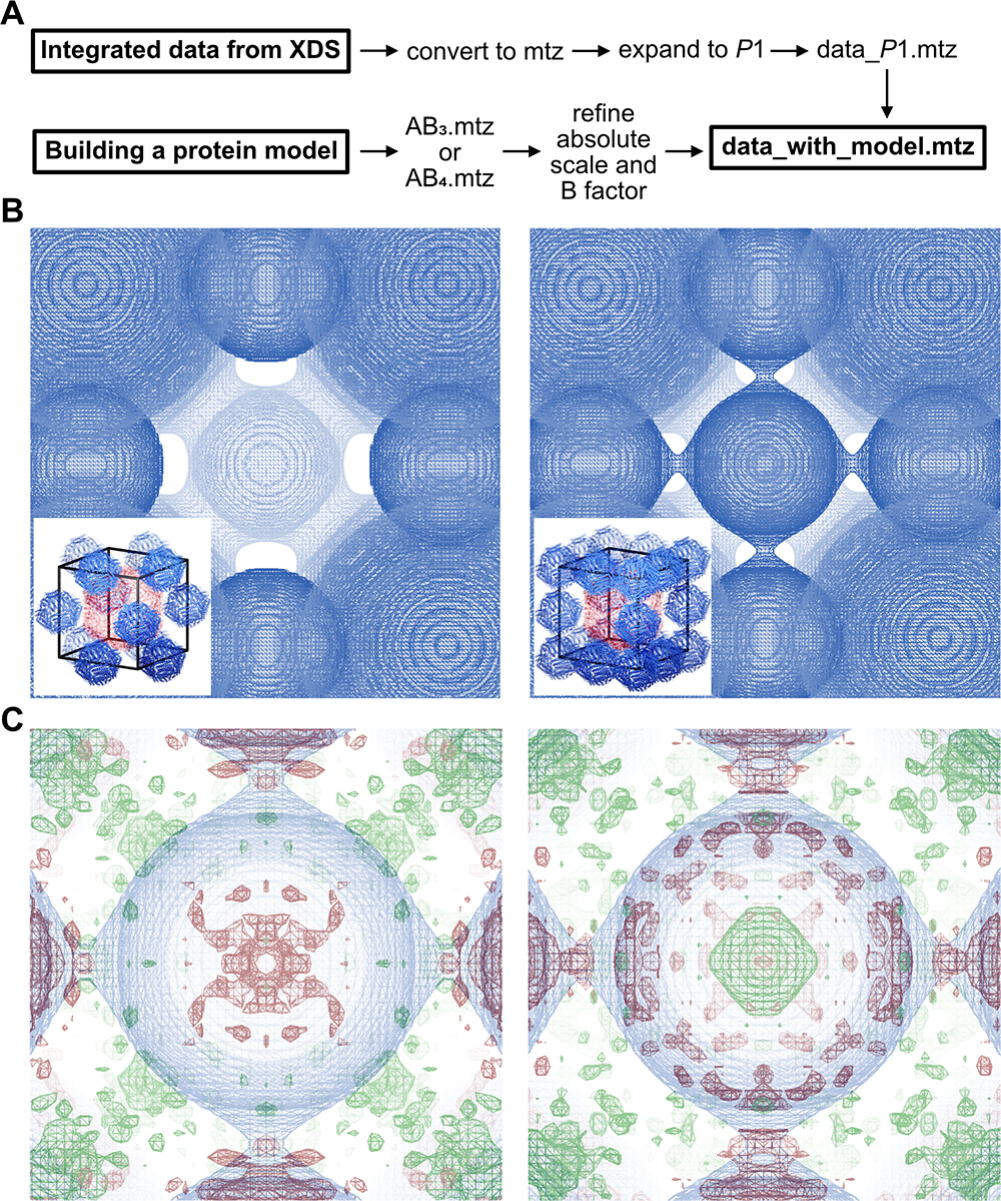
Comparison of structure models and differences in density maps of AB_3_ and AB_4_ structures. (A) Workflow of data processing and refinement. (B) The electron density maps of the two models are depicted. For each model, the AB_3_ (left) or AB_4_ (right) unit cell is shown (inset). For comparison, the density is visualized at an r.m.s.d. of −0.207 e^–^/nm^3^ for the AB_3_ model and −0.220 e^–^/nm^3^ for the AB_4_ model. (C) Difference densities for the two structural types. The difference electron density for the AB_3_ data was overlaid with the density map of the AB_4_ model (left). Very little electron difference density (green) is observed around the protein shell, indicating that further density (= ferritin cage) is present. For the right panel, refinement with the AB_4_ model shows a lot of negative electron density (red) for the position of the fourth ferritin, indicating that too much electron density is present in the model. Difference electron density visualized at an r.m.s.d. at 1.6 e^–^/nm^3^.

Although the averaged data shown in Figure 5 already give valuable insights into the structural composition, the simulated data are based on a rather coarse model: The diffraction data were generated with a software for powder diffraction of atomic structures, not nanoparticles (for details, see the Supporting Information). With such as simulation, the peak positions can be accurately predicted, but not the peak intensities. Therefore, we wondered if we could create a proper crystallographic model for the electron density within the unit cell and use the integrated data from the nanoparticle lattice diffraction (SC-SAXD) directly to refine this model against these data. Toward this end, a crystallographic model for the AuEnc^(neg)^/eFtn^(pos)^ sample was developed that contained both protein cages and nanoparticles (see details of construction in the Supporting Information and Figure S25). The obtained models were further processed (Figure 6A), and four parameters were refined against the experimental data. Interestingly, refinement *R* factors as low as 31.9 and 33.9% for the AB_3_ and AB_4_ models to 30 Å resolution over 85 unique reflections in the space group *P*432 were obtained, respectively.

Moreover, the difference density calculated for the AB_3_ model showed very little positive (green) density (Figure 6C) at the position of the fourth ferritin cage (not present in the AB_3_ model). This finding is in line with the difference density obtained for the AB_4_ model, which showed negative (red) features, indicating that the fourth ferritin is not occupied in this structure. The refinement statistics (Tables S12 and S13) further support the AB_3_-type structure, which yielded a lower *R* factor. In conclusion, these findings support the arrangement of the ferritin nanocages. Interestingly, with a ratio of 3:1, a cubic AB_3_ assembly type is known for inorganic compounds. This assembly is also referred to as the Cu_3_Au structure, because Cu_3_Au (auricupride) is the prototype compound for such structures.^48−50^ The Cu_3_Au structure can be observed for several AB_3_ compounds, such as for Mn_3_Pt^51^ alloys. Furthermore, nanoparticle superlattices with AB_3_ lattices have been synthesized.^52,53^

## Conclusions

In summary, we showed that two protein cages with distinct size, symmetry, and charge are suitable building blocks for biohybrid materials with a particular type of assembly. Several methods were employed to gain a detailed understanding of the arrangement of the two cages within the lattice. We encapsulated two types of nanoparticles into the ferritin and the encapsulin cage to enhance contrast in electron microscopy and scattering power in diffraction experiments. These composites were successfully assembled into binary 3D crystals. The separate and independent loading of each cage type enabled the determination of each sublattice type separately. Toward this end, we used SEM to visualize a simple cubic arrangement of the encapsulin cages within the binary lattice. To determine the ferritin positions, we used cryo-plasma-FIB to mill thin lamellae from a 3D crystal, suitable for electron cryo-tomography analysis of the ferritin sublattice. The structural type was elucidated as AB_3_. Single-crystal small-angle X-ray diffraction (SC-SAXD) was performed on crystals loaded with nanoparticles. Here, we used a unique approach of building a model containing both nanoparticles and protein cages. This model was refined against the single-crystal SAXD data and confirmed an assembly type of an AB_3_ structure.

The assemblies created in this study further expand the repertoire of available protein scaffolds for nanoparticle assembly. Because the supercharged protein cage assembly is driven by electrostatic interactions, the symmetry mismatch of the two different cage types can be overcome. By incorporation of the encapsulin cage, larger nanoparticles can be included, surpassing the limitations of existing protein cage assemblies. This potentially enables 3D lattices with enhanced plasmonic interactions between larger particles. Thus, the assembly created here provides opportunities for tailoring material properties and functionalities of hybrid materials based on nanoparticles and proteins. Importantly, using nanoparticles as marker particles enables the determination of structural details of crystalline protein-based matrices. Here, a heterobinary protein cage assembly has been formed based on two distinct building blocks. Interestingly, for inorganic compounds, even more complex structures involving three different building blocks are well known. Further extending crystalline biohybrid materials toward three distinct building blocks could be the next step toward multifunctional materials.

## Experimental Methods

### General Wet Lab Work

All chemicals were obtained from commercial sources and used without further purification. All solutions were prepared using ultrapure water prepared with a Purelab Flex 2 system (resistivity, 18.2 MΩ cm) manufactured by ELGA LabWater. Glassware and magnetic stir bars used for gold nanoparticle synthesis were cleaned with aqua regia and rinsed with ultrapure water to remove residual adsorbents.

### Gold Nanoparticle Synthesis

AuNPs were synthesized following the protocol of Schulz et al.^54^ Trisodium citrate dihydrate (121.0 mg) and citric acid (29.0 mg) were dissolved in 150 mL of ultrapure water. The citrate buffer was stirred using a 3 cm stir bar at 300 rpm and heated until boiling. To prevent excessive evaporation, the flask was covered with a small beaker. Separately, the gold precursor tetrachloroauric(III) acid trihydrate (16.0 mg) was dissolved in 50 mL of ultrapure water and heated to 80 °C. After boiling the citrate buffer for 14 min, EDTA (1.5 mg) was dissolved in 0.1 mL of ultrapure water and added to the solution. One additional minute later, the hot gold precursor solution was rapidly added to the reaction mixture. Upon a color change from colorless to wine-red, the mixture was continuously stirred and heated for an additional 20 min. Finally, the reaction mixture was cooled to room temperature and stored at 4 °C until further treatment.

### Gold Nanoparticle Ligand Exchange

The ligand exchange of citrate-stabilized AuNPs was conducted following a previously described procedure.^35^ For the ligand exchange from citrate to 11-(mercaptoundecyl)-*N*,*N*,*N*-trimethylammonium bromide (MUTAB), the latter was dissolved in a 100-fold excess with respect to the maximum number of ligands on the nanoparticle surface in a 2 M HCl solution. The MUTAB-containing HCl solution was then added to the NP solution, resulting in a HCl concentration of 0.1 M. The sample was incubated at room temperature for 48 h. To remove excess MUTAB and exchanged citrate, centrifugal concentration steps were performed. Initially, the sample was washed five times with 0.1 M HCl, followed by five washing steps with ultrapure water. Finally, the sample was concentrated to a volume of 1 mL.

Before the CLP functionalization, a stock solution of CLP (0.5 mg/mL in DMF) was prepared. The MUTAB-stabilized AuNPs were diluted 1:10 with DMF. Subsequently, an amount of CLP corresponding to 20 peptides per NP was added to the NP solution. After 16 h of incubation at room temperature, the NP solution was diluted 1:10 with water. The resulting solution was then concentrated and washed five times with water using a centrifugal filter and finally concentrated to a volume of 1 mL.

### Encapsulation of Gold Nanoparticles

The disassembly of encapsulin was achieved by subjecting 1.0 mg of Enc^(neg)^ (52.2 μL, from a 19.2 mg/mL stock solution) in storage buffer (20 mM Tris, pH 7.5, 0.3 M NaCl) to a 10-fold dilution with 10 mM phosphate pH 1.0 for 1 h at 4 °C. Reassembly was initiated by further diluting the sample 100-fold with reassembly buffer (20 mM phosphate, pH 7.0), adjusting the NaCl concentration toward 0.35 M with a 5 M NaCl solution and incubating it at room temperature overnight.

During the initiation of protein cage reassembly, the NP solution was added dropwise and gently swirled. After overnight incubation at room temperature, the sample was concentrated using an Amicon Ultra-15 centrifugal filter unit (100 kDa MWCO). The protein sample was first purified via ion-exchange (5 mL HiTrap Q HP anion exchange column, Cytiva) to separate protein from free nanoparticles. Subsequently, protein aggregates are removed from the monomeric protein cage sample through SEC (elution volume between 12 and 13 mL, Superose 6 Increase 10/300 GL gel filtration column, Cytiva). Absorbance at 520 nm is monitored to track the plasmonic gold nanoparticle absorption.

### Synthesis of Cerium Oxide Nanoparticles

The synthesis of cerium oxide nanoparticles was carried out as previously described.^29^ Buffer solutions for Ftn^(pos)^ (50 mM Tris, pH 7.5, 1 M NaCl) along with ultrapure water and H_2_O_2_ solutions were depleted of O_2_ by bubbling N_2_ through the solutions for at least 15 min. The buffer was then preheated in a 65 °C oil bath for 20 min in an oxygen-free atmosphere inside a two-necked round-bottom flask (25 mL) with constant stirring. Subsequently, 15 mg of Ftn^(pos)^ in buffer was added to a total volume of 20 mL. After 10 min, H_2_O_2_ (15 mM) and CeCl_3_ (30 mM) dissolved in deaerated ultrapure water were added in equal volumes using Perfusor compact S syringe pumps (B. Braun). A total of 37 Ce(III) ions were injected per ferritin cage, resulting in 2225 ions per cage. Once the addition was complete, the solution was kept in the water bath for another 15 min, and finally, 1.2 mL of EDTA (500 mM stock solution) was added at the end of the reaction. After an additional 15 min at 65 °C, the solution was centrifuged for 10 min at 14,000*g* and 4 °C. The supernatant was then rebuffered five times with buffer using an Amicon Ultra-15 centrifugal filter unit (30 kDa MWCO) to remove excess reagents and subsequently injected onto a HiLoad 16/600 Superdex 200 PG gel filtration column (Cytiva).

### Crystallization of the Two Protein Cages

Crystallization screening of Enc^(neg)^ and Ftn^(pos)^ was performed in a standard sitting drop vapor diffusion setup and further optimized using a hanging drop setup. A standard experiment utilized 24-well plates with a 500 μL reservoir solution and droplets of 4 μL in size, placed on siliconized glass cover slides, composed of 2:1:1 reservoir solution, Ftn^(pos)^, and Enc^(neg)^. Solutions were added to the droplet in this specific order. Crystallization condition contained 0.16 M ammonium sulfate in filtered ultrapure water. Protein stock solutions were used at a concentration of 4 mg/mL if not stated otherwise. Protein buffer was the respective SEC buffer: Enc^(neg)^: 20 mM Tris, pH 7.5, 0.3 M NaCl; Ftn^(pos)^: 50 mM Tris, pH 7.5, 1.0 M NaCl.

Plates were incubated in a temperature-controlled cabinet at 20 °C. Crystal growth was checked daily and formed crystals photographed under a Leica S9D microscope equipped with a FlexaCam C1 (Leica) or CrysCam Digital Microscope (Dunn Labortechnik).

### Stabilization of Protein Crystals

The cross-linker sulfosuccinimidyll-4-(*N*-maleimidomethyl)cyclohexan-1-carboxylat (Sulfo-SMCC) was used to stabilize protein crystals. A fresh stock solution of Sulfo-SMCC (4.8 mg/mL) was prepared with ultrapure water. Then, the cover slide with the drop containing crystals was briefly removed to add 100 μL of the freshly prepared stock solution to the crystallization condition and mix it. Depending on the crystal drop size, half the volume of drop is taken from the mixed reservoir and added for crystal cross-linking. The well was sealed with the cover slide, and crystals were cross-linked for 16 h at 20 °C.

### Single-Crystal X-ray Experiments

Protein crystals (empty cages and samples with cages loaded with nanoparticles) were soaked for 30 s in a solution containing 2 μL of 50% (v/v) glycerol and 2 μL of the ammonium sulfate reservoir solution, prior to vitrification in liquid nitrogen.

Diffraction data were collected at 100 K either at the P11 (DESY) or P14 (EMBL) beamline in Hamburg, Germany. Data were processed and scaled with XDS.^55^ Manual rebuilding of mutated residues, placing of water molecules and metal ions, and subsequent iterations of refinements were not possible due to low resolution for the data of the empty cages (Figure 1).

For the crystals with cages loaded with nanoparticles, the data were processed and scaled with XDS as for the empty cages. The indexed reflections were averaged to yield a peak for each reflection, and they are plotted in Figure 5 (black lines). For details on simulated diffraction data, please refer to the Supporting Information.

### Batch Crystallization

Crystals composed of Enc^(neg)^ and Ftn^(pos)^ were prepared via batch crystallization. For each protein, 50 μL of 12 mg/mL stock solutions was mixed and gently vortexed in a 1.5 mL tube, while 100 μL of a 0.08 M ammonium sulfate solution was added dropwise under gentle vortex (final volume, 200 μL). The tube containing the crystallization solution was incubated for 2 days at 20 °C in an incubator.

### Small-Angle X-ray Scattering

Protein crystals (empty Enc^(neg)^ and Ftn^(pos)^ cages) were stabilized by using Sulfo-SMCC. The crystal batch was prepared as described above and centrifuged for 2 min at 0.1 g to pellet the crystals. One hundred microliters of the supernatant was mixed with 1.4 mg of Sulfo-SMCC and supplemented with 300 μL of ultrapure water. Two hundred microliters of this Sulfo-SMCC stock solution was added into the batch. The crystals and solution were gently shaken and incubated for 16 h at 20 °C. Afterward, the cross-linked crystals were washed. First, the tube was centrifuged for 2 min at 1000*g* to remove the supernatant. To wash the crystals and remove the residual cross-linker, 300 μL of ultrapure water was added. After resuspension of the crystals, the process was repeated four times. Then, the crystals were stored at 20 °C until further usage.

For SAXS experiments, crystals were transferred into a Kapton capillary (Goodfellow Cambridge Ltd., England). The capillary was mounted onto a capillary holder. No rotation was performed due to the high amount of the sample. SAXS data were collected with a Dectris Eiger2 X 9M at beamline P62 (DESY, Germany) at 12 keV (1.0332 Å). The measurement was carried out at room temperature. The sample-to-detector distance was 6.24 m. A silver behenate standard sample was used for the calibration of the length of the scattering vector *q*. Azimuthal averaging of the 2D scattering data results in 1D SAXS data (Figure 1). More details on data processing are available in the Supporting Information.

### Transmission Electron Microscopy

In general, carbon-coated copper grids with a mesh size of 400 (Ted Pella, 01814-F-X) were utilized for transmission electron microscopy (TEM) measurements. Protein or nanoparticle-containing samples were analyzed by Stefan Werner (University of Hamburg, Germany) using a JEOL JEM 1011 operating at 100 kV. Unstained samples were prepared by drying 2 μL of the sample on the TEM grid. For uranyl acetate-stained samples, a 2% solution was used. Initially, the grid was incubated for 1 min on a droplet of 10 μL sample. Subsequently, the grid was washed three times in ultrapure water, followed by one wash and one incubation step (60 s) on a 2% uranyl acetate droplet. Any excess solution was blotted, and the grid was dried. Protein crystals were investigated using a FEI Tecnai G2 Spirit TWIN at 120 kV. Ten microliters of ultrapure water was placed on a TEM grid, and the crystal was transferred into the droplet before removing the droplet.

Image analysis was performed using ImageJ software. The size of AuNPs was determined by converting the images into binary images (black/white) using the threshold function to facilitate automatic counting and area determination. At least 200 NPs were considered for size determination. Protein cages were measured manually by analyzing 100 particles per sample. A circle was drawn around each protein cage to determine its size.

### Scanning Electron Microscopy

Protein crystals were washed with ultrapure water, transferred into a drop of ultrapure water on a silicon wafer, and dried under air. The dried protein crystals on the silicon wafer were imaged with a SCIOS FIB-SEM in SEM mode at varying acceleration voltages between 1 and 20 kV using either the Everhart-Thornley (ETD) detector for secondary electrons (SE) or the high-resolution through-lens detector in the SE mode.^42^ Cross sections were prepared on crystals with a tilt of 52° such that the Ga ion source is directed normal to the sample surface. In consecutive steps, the material was removed and the cross-section surface prepared using a final low current polishing. Cross-sectional SEM images were obtained with the ETD SE detector and the through-lens detector operated in SE mode.

### Preparation of Crystalline Lamellae by Xenon-Milling in Arctis Cryo-pFIB-SEM

Protein crystals were grown as described above (see Crystallization of the Two Protein Cages). We took a 7.5 μL solution containing washed cross-linked crystals and transferred it onto a freshly glow-discharged holey carbon grid (R 1.2/1.3 Cu200). Grids were left to sediment for 2–3 min and checked for the crystal presence on the carbon film by optical microscopy. Blotting was performed manually from both sides of each grid. Crystals were vitrified by plunging into liquid ethane-propane using a Leica GP2 plunger and transferred to an Arctis PlasmaFIB/SEM for milling under cryo-conditions as reported previously.^47^ A crystal for milling, ideally free of small ice contamination and in the center of the grid mesh, was identified in low-magnification SEM and FIB imaging. Crystals like these were easily found on every TEM grid tested, allowing the milling of several lamellae on each grid.

For lamella preparation, a focused beam of xenon ions accelerated at 30 kV was used. All milling steps were performed fully automated with the TFS WebUI milling application using a modified template for conventional lamella preparation. This protocol also includes application of a sandwich of a metallic platinum layer (12 kV, 0.15 μA beam current, 2 min), organoplatinum layer applied by the gas injection system (50 s), and another metallic platinum layer. After finding electron-to-ion beam crossover *z*-height and the milling angle with a target of 12°, stress relief cuts were milled on each side of the crystal with a beam current of 1 nA. The crystal was initially rough-milled using a beam current of 1 nA to ablate unwanted material and prepare an initial lamella with a thickness of approximately 1 μm. Subsequent reductions in the size of the crystal lamella came by further ablating crystalline material with reduced currents (medium milling at 0.3 nA, fine milling at 0.1 nA) above and below the initially milled volume with reduced current in a step-by-step fashion. The lamella thickness in each milling step was defined by a height overlap of 5 in each step. After fine milling of all lamellae, the final polishing step was performed with a target thickness of 230 nm. The final thickness and smoothness of the lamella were monitored with SEM operated at 2 kV and 25 pA. To prevent charging artifacts during TEM imaging, girds were platinum-coated once again as a final step (12 kV, 0.15 μA beam current, 5 s).

Following milling, the grids were directly transferred in the same grid cassette to the transmission electron microscope for tomographic data collection. The transfer from the autoloader to the autoloader system allows perfect orientation of the grid on the microscope stage with the milling direction of the grid perpendicular to the tilt axis. Crystalline lamellae were located in low-magnification images montaged to a grid map. Each milled lamella was clearly visible compared to an unmilled crystal, which appeared dark.

Tomograms were collected on a Titan Krios G3 (ThermoFisher Scientific) transmission electron microscope, operated at 300 kV and equipped with a Gatan Bioquantum energy filter operated in zero-loss mode (20 eV energy slit width). Images were acquired on a Gatan K3 electron counting direct detection camera (Gatan Inc.) in dose fractionation mode using SerialEM software^56^ at a nominal magnification of 42,000× (physical pixel size, 0.21 nm * 0.21 nm/px) and on-the-fly frame alignment using the SerialEM plugin. Tilt series acquisition was performed in the dose-symmetric tilt scheme^57^ with a total dose of 100 e/A^2^ evenly distributed over 41 tilt images acquired in dose fractionation mode with an increment of 2°.

Tilt stacks were reconstructed into tomogram volumes at a binning factor of 4 using patch tracking alignment and weighted back projection in imod software package version 4.11.1.^58^ The tomogram was visualized with ChimeraX.^59^ The tomogram was oriented parallel to one lattice plane of encapsulin cages (rotate slab). For analyzing the sublattices, spheres with the same diameter as the protein cages (12 nm vs 24 nm) were placed into the tomogram. First, marker spheres were placed. With these markers and the spacing observed in the tomogram, the sublattices for the protein cages were created using the lattice command, a Python script developed by Tom Goddard for this purpose.

### Structure Modeling and Refinement for Single-Crystal Small-Angle X-ray Diffraction (SC-SAXD) Data

In order to generate a crystallographic model of the electron density, a low-resolution unit cell was constructed according to the following steps. A cubic grid was constructed with a unit cell dimension of 244.5 Å and a grid of 128 × 128 × 128 voxels. Smooth spheres of density were generated by specifying a maximum radius *M* beyond which density was set to zero and a minimum radius *m* where the density was set to one. The density in each intervening voxel was calculated according to the following equation, where ρ_*xyz*_ is the density at voxel (*x*, *y*, *z*) in the map and r_*xyz*_ is the magnitude of the real space vector from the origin to voxel (*x*, *y*, *z*):
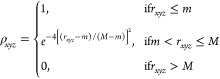


This is used to generate starting maps, which are operated on in order to build a model of the encapsulin, gold nanoparticle, and ferritin components. The operation workflow for generating this map is shown in Figure S25, and spheres were calculated from the known geometry of encapsulin and ferritin molecules and not refined against experimental data (see below). Several minimum and maximum radii of the gold nanoparticle were tested, 55 to 75 Å, 50 to 80 Å, 40 to 90 Å, and 30 to 100 Å, and from those, 40 to 90 Å was manually determined to be the best fit against experimental data. This workflow generates either the AB_3_ or AB_4_ model for further analysis. The Fourier transform of these models were stored in MTZ format.

Integrated and merged reflections with XDS were converted to MTZ format and expanded to P1 using sftools.^60^ An absolute scale and B factor were manually refined using sftools to optimize the *R* factor against the experimental data. Electron density *F*_o_ – *F*_c_ difference maps were calculated for each of the AB_3_ and AB_4_ models.

## Supporting Information Available

Additional details on experimental methods and/or supporting figures and tables for protein mutagenesis, protein production and purification, fast protein liquid chromatography, sodium dodecyl sulfate gel electrophoresis, dynamic light scattering, zeta potential measurements, small-angle X-ray scattering and diffraction, electrospray ionization mass spectrometry, atomic force microscopy, scanning transmission electron microscopy, electron cryo-tomography, and transmission electron microscopy (PDF)

## Notes

An earlier version of this manuscript was deposited as a preprint: Beck, T.; Rütten, M.; Lang, L.; Wagler, H.; Lach, M.; Keller, T.; Stierle, A.; Ginn, H., Assembly of differently sized supercharged protein nanocages into superlattices for construction of binary nanoparticle–protein materials. 2024. ChemRxiv. 10.26434/chemrxiv-2024-m179z (accessed August 14, 2024).

## References

[ref1] Zhu K.; Ju Y.; Xu J.; Yang Z.; Gao S.; Hou Y. Magnetic Nanomaterials: Chemical Design, Synthesis, and Potential Applications. Acc. Chem. Res. 2018, 51, 404–413. 10.1021/acs.accounts.7b00407. 29412634

[ref2] Ulijn R. V.; Smith A. M. Designing Peptide Based Nanomaterials. Chem. Soc. Rev. 2008, 37, 664–675. 10.1039/b609047h. 18362975

[ref3] You H.; Yang S.; Ding B.; Yang H. Synthesis of Colloidal Metal and Metal Alloy Nanoparticles for Electrochemical Energy Applications. Chem. Soc. Rev. 2013, 42, 2880–2904. 10.1039/C2CS35319A. 23152097

[ref4] Kan S.; Mokari T.; Rothenberg E.; Banin U. Synthesis and Size-Dependent Properties of Zinc-Blende Semiconductor Quantum Rods. Nat. Mater. 2003, 2, 155–158. 10.1038/nmat830. 12612671

[ref5] Jia C.-J.; Sun L.-D.; Luo F.; Han X.-D.; Heyderman L. J.; Yan Z.-G.; Yan C.-H.; Zheng K.; Zhang Z.; Takano M.; Hayashi N.; Eltschka M.; Kläui M.; Rüdiger U.; Kasama T.; Cervera-Gontard L.; Dunin-Borkowski R. E.; Tzvetkov G.; Raabe J. Large-Scale Synthesis of Single-Crystalline Iron Oxide Magnetic Nanorings. J. Am. Chem. Soc. 2008, 130, 16968–16977. 10.1021/ja805152t. 19053430

[ref6] Yeh C.-Y.; Lu Z. W.; Froyen S.; Zunger A. Zinc-Blende--Wurtzite Polytypism in Semiconductors. Phys. Rev. B 1992, 46, 10086–10097. 10.1103/PhysRevB.46.10086. 10002848

[ref7] Dshemuchadse J.; Steurer W. Some Statistics on Intermetallic Compounds. Inorg. Chem. 2015, 54, 1120–1128. 10.1021/ic5024482. 25470110

[ref8] Chu Z.; Chu X.; Zhao Y.; Ye Q.; Jiang J.; Zhang X.; You J. Emerging Low-Dimensional Crystal Structure of Metal Halide Perovskite Optoelectronic Materials and Devices. Small Struct. 2021, 2, 2000133 10.1002/sstr.202000133.

[ref9] Andersen H. L.; Saura-Múzquiz M.; Granados-Miralles C.; Canévet E.; Lock N.; Christensen M. Crystalline and Magnetic Structure–Property Relationship in Spinel Ferrite Nanoparticles. Nanoscale 2018, 10, 14902–14914. 10.1039/C8NR01534A. 30044457

[ref10] Zhong Lin W. Zinc Oxide Nanostructures: Growth, Properties and Applications. J. Phys.: Condens. Matter 2004, 16, R829 10.1088/0953-8984/16/25/R01.

[ref11] Morris E.; Groy T.; Leinenweber K. Crystal Structure and Bonding in the High-Pressure Form of Fluorite (Caf2). J. Phys. Chem. Solids 2001, 62, 1117–1122. 10.1016/S0022-3697(00)00291-2.

[ref12] Guo T.; Lin M.; Huang J.; Zhou C.; Tian W.; Yu H.; Jiang X.; Ye J.; Shi Y.; Xiao Y.; Bian X.; Feng X. The Recent Advances of Magnetic Nanoparticles in Medicine. J. Nanomater. 2018, 2018, 1–8. 10.1155/2018/7805147.

[ref13] Daniel M. C.; Astruc D. Gold Nanoparticles: Assembly, Supramolecular Chemistry, Quantum-Size-Related Properties, and Applications toward Biology, Catalysis, and Nanotechnology. Chem. Rev. 2004, 104, 293–346. 10.1021/cr030698+. 14719978

[ref14] Kang K. A.; Wang J.; Jasinski J. B.; Achilefu S. Fluorescence Manipulation by Gold Nanoparticles: From Complete Quenching to Extensive Enhancement. J. Nanobiotechnology 2011, 9, 16 10.1186/1477-3155-9-16. 21569249 PMC3112388

[ref15] Matricardi C.; Hanske C.; Garcia-Pomar J. L.; Langer J.; Mihi A.; Liz-Marzan L. M. Gold Nanoparticle Plasmonic Superlattices as Surface-Enhanced Raman Spectroscopy Substrates. ACS Nano 2018, 12, 8531–8539. 10.1021/acsnano.8b04073. 30106555

[ref16] Raino G.; Becker M. A.; Bodnarchuk M. I.; Mahrt R. F.; Kovalenko M. V.; Stoferle T. Superfluorescence from Lead Halide Perovskite Quantum Dot Superlattices. Nature 2018, 563, 671–675. 10.1038/s41586-018-0683-0. 30405237

[ref17] Li J.; Wang Y.; Zhou T.; Zhang H.; Sun X.; Tang J.; Zhang L.; Al-Enizi A. M.; Yang Z.; Zheng G. Nanoparticle Superlattices as Efficient Bifunctional Electrocatalysts for Water Splitting. J. Am. Chem. Soc. 2015, 137, 14305–14312. 10.1021/jacs.5b07756. 26496655

[ref18] Kalsin A. M.; Fialkowski M.; Paszewski M.; Smoukov S. K.; Bishop K. J.; Grzybowski B. A. Electrostatic Self-Assembly of Binary Nanoparticle Crystals with a Diamond-Like Lattice. Science 2006, 312, 420–4. 10.1126/science.1125124. 16497885

[ref19] Weidman M. C.; Nguyen Q.; Smilgies D.-M.; Tisdale W. A. Impact of Size Dispersity, Ligand Coverage, and Ligand Length on the Structure of Pbs Nanocrystal Superlattices. Chem. Mater. 2018, 30, 807–816. 10.1021/acs.chemmater.7b04322.

[ref20] Young K. L.; Ross M. B.; Blaber M. G.; Rycenga M.; Jones M. R.; Zhang C.; Senesi A. J.; Lee B.; Schatz G. C.; Mirkin C. A. Using DNA to Design Plasmonic Metamaterials with Tunable Optical Properties. Adv. Mater. 2014, 26, 653–659. 10.1002/adma.201302938. 24166990

[ref21] Si K. J.; Chen Y.; Shi Q.; Cheng W. Nanoparticle Superlattices: The Roles of Soft Ligands. Adv. Sci. 2018, 5, 1700179 10.1002/advs.201700179. PMC5770676 29375958

[ref22] Lewis D. J.; Carter D. J. D.; Macfarlane R. J. Using DNA to Control the Mechanical Response of Nanoparticle Superlattices. J. Am. Chem. Soc. 2020, 142, 19181–19188. 10.1021/jacs.0c08790. 33140957

[ref23] Tian Y.; Zhang Y.; Wang T.; Xin H. L.; Li H.; Gang O. Lattice Engineering through Nanoparticle–DNA Frameworks. Nat. Mater. 2016, 15, 654–661. 10.1038/nmat4571. 26901516 PMC5282967

[ref24] Mueller N. S.; Okamura Y.; Vieira B. G. M.; Juergensen S.; Lange H.; Barros E. B.; Schulz F.; Reich S. Deep Strong Light–Matter Coupling in Plasmonic Nanoparticle Crystals. Nature 2020, 583, 780–784. 10.1038/s41586-020-2508-1. 32728238

[ref25] Kalsin A. M.; Fialkowski M.; Paszewski M.; Smoukov S. K.; Bishop K. J. M.; Grzybowski B. A. Electrostatic Self-Assembly of Binary Nanoparticle Crystals with a Diamond-Like Lattice. Science 2006, 312, 420–424. 10.1126/science.1125124. 16497885

[ref26] Pillai P. P.; Kowalczyk B.; Grzybowski B. A. Self-Assembly of Like-Charged Nanoparticles into Microscopic Crystals. Nanoscale 2016, 8, 157–61. 10.1039/C5NR06983A. 26616821

[ref27] Pinkard A.; Champsaur A. M.; Roy X. Molecular Clusters: Nanoscale Building Blocks for Solid-State Materials. Acc. Chem. Res. 2018, 51, 919–929. 10.1021/acs.accounts.8b00016. 29605996

[ref28] Edwardson T. G. W.; Levasseur M. D.; Tetter S.; Steinauer A.; Hori M.; Hilvert D. Protein Cages: From Fundamentals to Advanced Applications. Chem. Rev. 2022, 122, 9145–9197. 10.1021/acs.chemrev.1c00877. 35394752

[ref29] Huard D. J.; Kane K. M.; Tezcan F. A. Re-Engineering Protein Interfaces Yields Copper-Inducible Ferritin Cage Assembly. Nat. Chem. Biol. 2013, 9, 169–76. 10.1038/nchembio.1163. 23340339

[ref30] Uchida M.; McCoy K.; Fukuto M.; Yang L.; Yoshimura H.; Miettinen H. M.; LaFrance B.; Patterson D. P.; Schwarz B.; Karty J. A.; Prevelige P. E. Jr.; Lee B.; Douglas T. Modular Self-Assembly of Protein Cage Lattices for Multistep Catalysis. ACS Nano 2018, 12, 942–953. 10.1021/acsnano.7b06049. 29131580 PMC5870838

[ref31] Künzle M.; Eckert T.; Beck T. Metal-Assisted Assembly of Protein Containers Loaded with Inorganic Nanoparticles. Inorg. Chem. 2018, 57, 13431–13436. 10.1021/acs.inorgchem.8b01995. 30351078

[ref32] Künzle M.; Eckert T.; Beck T. Binary Protein Crystals for the Assembly of Inorganic Nanoparticle Superlattices. J. Am. Chem. Soc. 2016, 138, 12731–12734. 10.1021/jacs.6b07260. 27617514

[ref33] Han K.; Zhang Z.; Tezcan F. A. Spatially Patterned, Porous Protein Crystals as Multifunctional Materials. J. Am. Chem. Soc. 2023, 145, 19932–19944. 10.1021/jacs.3c06348. 37642457

[ref34] Junker N. O.; Lindenau A.; Rütten M.; Lach M.; Nedilko A.; Chigrin D. N.; von Plessen G.; Beck T. Optical Properties of Metacrystals Based on Protein Nanocages. Adv. Funct. Mater. 2023, 33, 2303260 10.1002/adfm.202303260.

[ref35] Lach M.; Strelow C.; Meyer A.; Mews A.; Beck T. Encapsulation of Gold Nanoparticles into Redesigned Ferritin Nanocages for the Assembly of Binary Superlattices Composed of Fluorophores and Gold Nanoparticles. ACS Appl. Mater. Interfaces 2022, 14, 10656–10668. 10.1021/acsami.1c20520. 35166537

[ref36] Ross M. B.; Ku J. C.; Vaccarezza V. M.; Schatz G. C.; Mirkin C. A. Nanoscale Form Dictates Mesoscale Function in Plasmonic DNA–Nanoparticle Superlattices. Nat. Nanotechnol. 2015, 10, 453–458. 10.1038/nnano.2015.68. 25867942

[ref37] Jones M. R.; Macfarlane R. J.; Lee B.; Zhang J.; Young K. L.; Senesi A. J.; Mirkin C. A. DNA-Nanoparticle Superlattices Formed from Anisotropic Building Blocks. Nat. Mater. 2010, 9, 913–917. 10.1038/nmat2870. 20890281

[ref38] Künzle M.; Mangler J.; Lach M.; Beck T. Peptide-Directed Encapsulation of Inorganic Nanoparticles into Protein Containers. Nanoscale 2018, 10, 22917–22926. 10.1039/C8NR06236F. 30499576

[ref39] Giessen T. W.; Orlando B. J.; Verdegaal A. A.; Chambers M. G.; Gardener J.; Bell D. C.; Birrane G.; Liao M.; Silver P. A. Large Protein Organelles Form a New Iron Sequestration System with High Storage Capacity. eLife 2019, 8, e46070 10.7554/eLife.46070. 31282860 PMC6668986

[ref40] Jones J. A.; Benisch R.; Giessen T. W. Encapsulin Cargo Loading: Progress and Potential. J. Mater. Chem. B 2023, 11, 4377–4388. 10.1039/D3TB00288H. 37158413 PMC10225969

[ref41] Toussaint L.; Bertrand L.; Hue L.; Crichton R. R.; Declercq J.-P. High-Resolution X-Ray Structures of Human Apoferritin H-Chain Mutants Correlated with Their Activity and Metal-Binding Sites. J. Mol. Biol. 2007, 365, 440–452. 10.1016/j.jmb.2006.10.010. 17070541

[ref42] LaFrance B. J.; Cassidy-Amstutz C.; Nichols R. J.; Oltrogge L. M.; Nogales E.; Savage D. F. The Encapsulin from Thermotoga Maritima Is a Flavoprotein with a Symmetry Matched Ferritin-Like Cargo Protein. Sci. Rep. 2021, 11, 22810 10.1038/s41598-021-01932-w. 34815415 PMC8610991

[ref43] Wiryaman T.; Toor N. Cryo-Em Structure of a Thermostable Bacterial Nanocompartment. IUCrJ. 2021, 8, 342–350. 10.1107/S2052252521001949. 33953921 PMC8086157

[ref44] Beck T.; Tetter S.; Künzle M.; Hilvert D. Construction of Matryoshka-Type Structures from Supercharged Protein Nanocages. Angew. Chem., Int. Ed. 2015, 54, 937–40. 10.1002/anie.201408677. 25392947

[ref45] Stierle A.; Keller T. F.; Noei H.; Vonk V.; Roehlsberger R. Desy Nanolab. JLSRF 2016, 2, A76 10.17815/jlsrf-2-140.

[ref46] Martynowycz M. W.; Gonen T. Protocol for the Use of Focused Ion-Beam Milling to Prepare Crystalline Lamellae for Microcrystal Electron Diffraction (Microed). STAR Protocols 2021, 2, 100686 10.1016/j.xpro.2021.100686. 34382014 PMC8339237

[ref47] Martynowycz M. W.; Shiriaeva A.; Clabbers M. T. B.; Nicolas W. J.; Weaver S. J.; Hattne J.; Gonen T. A Robust Approach for Microed Sample Preparation of Lipidic Cubic Phase Embedded Membrane Protein Crystals. Nat. Commun. 2023, 14, 1–15. 10.1038/s41467-023-36733-4. 36841804 PMC9968316

[ref48] Mehl M. J. A Brief History of Strukturbericht Symbols and Other Crystallographic Classification Schemes. J. Phys.: Conf. Ser. 2019, 1290, 012016 10.1088/1742-6596/1290/1/012016.

[ref49] Bragg W. L. Strukturbericht, 1913–1928. Nature 1931, 128, 511–512. 10.1038/128511a0.

[ref50] Owen E. A.; Liu Y. H. Xl. The Thermal Expansion of the Gold-Copper Alloy Aucu3. London, Edinburgh Dublin Philos. Mag. J. Sci. 1947, 38, 354–360. 10.1080/14786444708521607.

[ref51] An N.; Tang M.; Hu S.; Yang H.; Fan W.; Zhou S.; Qiu X. Structure and Strain Tunings of Topological Anomalous Hall Effect in Cubic Noncollinear Antiferromagnet Mn3 pt Epitaxial Films. Sci. China Phys., Mech. Astron. 2020, 63, 297511 10.1007/s11433-019-1525-6.

[ref52] Deng K.; Xu L.; Guo X.; Wu X.; Liu Y.; Zhu Z.; Li Q.; Zhan Q.; Li C.; Quan Z. Binary Nanoparticle Superlattices for Plasmonically Modulating Upconversion Luminescence. Small 2020, 16, 2002066 10.1002/smll.202002066. 32815270

[ref53] Shevchenko E. V.; Talapin D. V.; Kotov N. A.; O’Brien S.; Murray C. B. Structural Diversity in Binary Nanoparticle Superlattices. Nature 2006, 439, 55–9. 10.1038/nature04414. 16397494

[ref54] Schulz F.; Homolka T.; Bastus N. G.; Puntes V.; Weller H.; Vossmeyer T. Little Adjustments Significantly Improve the Turkevich Synthesis of Gold Nanoparticles. Langmuir 2014, 30, 10779–84. 10.1021/la503209b. 25127436

[ref55] Kabsch W. XDS. Acta. Crystallogr. 2010, D66, 125–132. 10.1107/S0907444909047337. PMC2815665 20124692

[ref56] Mastronarde D. N. Serialem: A Program for Automated Tilt Series Acquisition on Tecnai Microscopes Using Prediction of Specimen Position. Microsc. Microanal. 2003, 9, 1182–1183. 10.1017/S1431927603445911.

[ref57] Hagen W. J. H.; Wan W.; Briggs J. A. G. Implementation of a Cryo-Electron Tomography Tilt-Scheme Optimized for High Resolution Subtomogram Averaging. J. Struct Biol. 2017, 197, 191–198. 10.1016/j.jsb.2016.06.007. 27313000 PMC5287356

[ref58] Mastronarde D. N.; Held S. R. Automated Tilt Series Alignment and Tomographic Reconstruction in Imod. J. Struct Biol. 2017, 197, 102–113. 10.1016/j.jsb.2016.07.011. 27444392 PMC5247408

[ref59] Meng E. C.; Goddard T. D.; Pettersen E. F.; Couch G. S.; Pearson Z. J.; Morris J. H.; Ferrin T. E. UCSF ChimeraX: Tools for structure building and analysis. Protein Sci. 2023, 32, e4792 10.1002/pro.4792. 37774136 PMC10588335

[ref60] Agirre J.; Atanasova M.; Bagdonas H.; Ballard C. B.; Basle A.; Beilsten-Edmands J.; Borges R. J.; Brown D. G.; Burgos-Marmol J. J.; Berrisford J. M.; Bond P. S.; Caballero I.; Catapano L.; Chojnowski G.; Cook A. G.; Cowtan K. D.; Croll T. I.; Debreczeni J. E.; Devenish N. E.; Dodson E. J.; Drevon T. R.; Emsley P.; Evans G.; Evans P. R.; Fando M.; Foadi J.; Fuentes-Montero L.; Garman E. F.; Gerstel M.; Gildea R. J.; Hatti K.; Hekkelman M. L.; Heuser P.; Hoh S. W.; Hough M. A.; Jenkins H. T.; Jimenez E.; Joosten R. P.; Keegan R. M.; Keep N.; Krissinel E. B.; Kolenko P.; Kovalevskiy O.; Lamzin V. S.; Lawson D. M.; Lebedev A. A.; Leslie A. G. W.; Lohkamp B.; Long F.; Maly M.; McCoy A. J.; McNicholas S. J.; Medina A.; Millan C.; Murray J. W.; Murshudov G. N.; Nicholls R. A.; Noble M. E. M.; Oeffner R.; Pannu N. S.; Parkhurst J. M.; Pearce N.; Pereira J.; Perrakis A.; Powell H. R.; Read R. J.; Rigden D. J.; Rochira W.; Sammito M.; Sanchez Rodriguez F.; Sheldrick G. M.; Shelley K. L.; Simkovic F.; Simpkin A. J.; Skubak P.; Sobolev E.; Steiner R. A.; Stevenson K.; Tews I.; Thomas J. M. H.; Thorn A.; Valls J. T.; Uski V.; Uson I.; Vagin A.; Velankar S.; Vollmar M.; Walden H.; Waterman D.; Wilson K. S.; Winn M. D.; Winter G.; Wojdyr M.; Yamashita K. The Ccp4 Suite: Integrative Software for Macromolecular Crystallography. Acta Crystallogr. D 2023, 79, 449–461. 10.1107/S2059798323003595. PMC10233625 37259835

