# Supplementary material for: Assembly of Differently Sized Supercharged Protein Nanocages into Superlattices for Construction of Binary Nanoparticle–Protein Materials
Source: ACS Nano. 2024 Aug 27;18(36):25325–36. doi: 10.1021/acsnano.4c09551 (PMC11394343; doi:10.1021/acsnano.4c09551)
Supplement: Supplementary file 1 — nn4c09551_si_001.pdf [file nn4c09551_si_001.pdf]

Supporting Information

Assembly of differently sized supercharged protein nanocages into superlattices for construction of binary nanoparticle-protein materials

Michael Rütten[†], *Laurin Lang*[†,‡], *Henrike Wagler*[†], *Marcel Lach*[†], *Niklas Mucke*[†], *Ulrike Laugks*[≠,ᐩ,⊢], *Carolin Seuring*[≠,ᐩ,⊢,‡], *Thomas F. Keller*[§,⊥], *Andreas Stierle*[§,⊥], *Helen M. Ginn*[∥,≈], *Tobias Beck*[†,‡,*]

† Institute of Physical Chemistry, Department of Chemistry, University of Hamburg, 20146 Hamburg, Germany

‡ The Hamburg Centre for Ultrafast Imaging, University of Hamburg, Hamburg, Germany

≠ Centre for Structural Systems Biology (CSSB), 22607 Hamburg, Germany

ᐩ Department of Structural Cell Biology of Viruses, Leibniz Institute of Virology, 20251 Hamburg, Germany

⊢ Department of Chemistry, University of Hamburg, 20146 Hamburg, Germany

§ Centre for X-ray and Nano Science (CXNS), Deutsches Elektronen-Synchrotron DESY, 22607 Hamburg, Germany

⊥ Department of Physics, University of Hamburg, 22607 Hamburg, Germany

∥ Center for Free-Electron Laser Science (CFEL), Deutsches Elektronen-Synchrotron DESY, 22607 Hamburg, Germany

≈ Institute for Nanostructure and Solid State Physics, Department of Physics, University of Hamburg, 22761 Hamburg, Germany

Corresponding Author: tobias.beck@uni-hamburg.de

Table of contents

Supporting materials and methods ... 3
General wet lab work ... 3
Mutagenesis ... 3
Protein Production and Purification ... 4
Transformation ... 4
Precultures ... 4
Production Cultures ... 5
Protein Purification ... 5
Fast protein liquid chromatography – general purification procedures ... 6
Fast protein liquid chromatography – calibration and analysis ... 6
SDS gel electrophoresis – calibration and analysis ... 7
Dynamic light scattering (DLS) ... 8
Zeta (ζ) potential measurements ... 8
Small-angle X-ray scattering and diffraction ... 8
Electrospray ionization mass spectrometry ... 9
UV-Vis spectroscopy ... 10
Scanning transmission electron microscopy ... 10
Atomic force microscopy ... 11
Supporting Figures ... 12
Supporting Tables ... 26
References ... 31

List of figures

Figure S1: Stained TEM micrograph of Enc$^{(neg)}$ with histogram. 12
Figure S2: Protein purification of Enc$^{(neg)}$....................12
Figure S3: ESI MS spectra to characterize Enc$^{(neg)}$....................12
Figure S4: Size-exclusion runs of Enc$^{(neg)}$ for chromatography calibration....................13
Figure S5: Size-exclusion runs of Ftn$^{(pos)}$ for chromatography calibration....................14
Figure S6: Size-exclusion runs of all five batch samples. 14
Figure S7: Averaged SEC runs with corresponding calibration curves....................15
Figure S8: SDS gel and calibration curve....................16
Figure S9: SDS gels of batch crystallization samples. 16
Figure S10: TEM micrograph of CeFtn$^{(pos)}$. 17
Figure S11: FPLC SEC of cerium oxide nanoparticle loaded Ftn$^{(pos)}$. 17
Figure S12: TEM image of synthesized gold nanoparticles with histogram. 18
Figure S13: Purification of gold nanoparticle loaded Enc$^{(neg)}$. 18
Figure S14: TEM micrograph of AuNP-loaded Enc$^{(neg)}$. 19
Figure S15: Dynamic light scattering of the building blocks. 19
Figure S16: AFM images of hetero binary AuEnc$^{(neg)}$/eFtn$^{(pos)}$ crystal. 20
Figure S17: SEM image of a hetero binary AuEnc$^{(neg)}$/eFtn$^{(pos)}$ crystal with a cut cross-section prepared by ion milling. 21
Figure S18: STEM image of hetero binary AuEnc$^{(neg)}$/eFtn$^{(pos)}$ crystal....................21
Figure S19: AFM images of hetero binary eEnc$^{(neg)}$/CeFtn$^{(pos)}$ crystal. 22
Figure S20: SEM images of hetero binary eEnc$^{(neg)}$/CeFtn$^{(pos)}$ crystal. 22
Figure S21: SEM image of hetero binary eEnc$^{(neg)}$/CeFtn$^{(pos)}$ crystal before and after milling. 22
Figure S22. In situ structural characterization of empty Enc$^{(neg)}$ and CeO_2 NP-loaded Ftn$^{(pos)}$ by cryo transmission electron microscopy (cryo-TEM) at high magnification. 23
Figure S23: Electron cryo-tomogram of a lamella from an eEnc$^{(neg)}$/CeFtn$^{(pos)}$ crystal. 23
Figure S24: Comparison of an AB_3 and AB_4 unit cell model. 24
Figure S25: Schematic overview of structure modelling....................24
Figure S26: Representative X-ray diffraction image for the AuEnc$^{(neg)}$/eFtn$^{(pos)}$ crystal. 25

List of tables

Table S1: Sequence of primer used for the mutation of *T. maritima* wild type encapsulin....26
Table S2: Protein sequences for protein variants....................26
Table S3: Two-step PCR cycling protocol. 26
Table S4: Sequence of the cargo-loading peptide. 27
Table S5: Data points for FPLC calibration for both proteins. 27
Table S6: Averaged peak areas of protein samples for calibration. 27
Table S7: Evaluation of peak areas from batch samples via peak areas....................27
Table S8: Integrated density values for calibration samples on SDS gel. 28
Table S9: Integrated density values for five batch crystallization samples....................28
Table S10: Summary of FPLC and SDS data for ratio determination. 29
Table S11: Unit cell parameters from single crystal SAXD data of nanoparticle-loaded protein crystals 29
Table S12: Refinement statistics for single crystal SAXS data based on AB_3 model....................29
Table S13: Refinement statistics for single crystal SAXS data based on AB_4 model....................30

Supporting materials and methods

General wet lab work

All chemicals were obtained from commercial sources and used without further purification. All solutions are prepared using ultrapure water prepared with a Purelab Flex 2 system (resistivity 18.2 MΩ cm) manufactured by ELGA LabWater. Glassware and magnetic stir bars used for gold nanoparticle synthesis were cleaned with aqua regia and rinsed with ultrapure water to remove residual adsorbents. Prepared buffers were filtered with a 0.22 µm membrane filter (Carl Roth).

Mutagenesis

Enc$^{(neg)}$ containing a singular point mutation in comparison to wildtype encapsulin was made by performing QuikChange™ mutagenesis on the respective parent plasmid. Primer designed for the mutation W90E is shown in Table S1 and was ordered at Eurofins Genomics.

To start, a mixture was prepared by combining 2.9 µL of pET-22b(+) plasmid (7 ng/µL) containing the gene of interest, 1 µL of 10 mM dNTP mix, 5 µL of buffer (200 mM Tris-HCl pH 8.8, 100 mM ammonium sulfate, 100 mM KCl, 1 % (v/v) Triton X-100, 1 mg/mL BSA), 1 µL of Pfu DNA polymerase (2.5 U/µL), and 38.1 µL of ultrapure water. Next, the mixture was equally divided into two microtubes. In each tube, 1 µL of either forward or reverse primer (100 pmol/µL) was added. The PCR (Eppendorf Mastercycler Nexus PCR Cycler) was then carried out at specific parameters (Table S2). After segment 2 ("Hold"), both mixtures containing the forward and reverse primers were combined. After completing the PCR, the parental plasmid was digested by adding 1 µL of DpnI (10 U/µL) and incubating overnight at 37 °C. The following day, DpnI was deactivated by heating at 80 °C for 20 minutes, and the reaction was purified using the NucleoSpin Gel and PCR Clean-up Kit (Macherey-Nagel) following the manufacturer's protocol. Next, 100 µL of competent cells were mixed with 200 ng of the plasmid and incubated on ice for 30 minutes, followed by a heat shock at 42 °C for 45 seconds. After two minutes on ice, the competent cells were added to 0.9 mL of super optimal broth (SOB) media and incubated at 37 °C, shaking at 250 rpm, for 1 hour. The cells were then gently pelleted by centrifugation at 1000 g for 1 minute. Subsequently, the cells were resuspended in 100 µL of media, plated onto LB agar plates with ampicillin (150 µg/mL), and incubated for 16 hours at 37 °C. The next day, a single colony was picked and incubated at 37 °C and 250 rpm in 5 mL of sterile LB medium supplemented with 150 µg/mL ampicillin.

After 16 hours of incubation, the plasmids were extracted using the NucleoSpin Plasmid Miniprep Kit (Macherey-Nagel).

DNA sequencing was carried out at Eurofins Genomics. 500 ng Plasmid were mixed with 25 pmol T7 forward or reverse primer in a 10 µL solution.

Protein Production and Purification

Production of Enc$^{(neg)}$ and Ftn$^{(pos)}$ is identical to the production of wildtype encapsulin and Ftn$^{(pos)}$ previously published with minor adjustments.[1-2]

Transformation

Calcium-competent *E.Coli* cells (100 uL stocks) were retrieved from -80 °C and allowed to equilibrate on ice for 10 minutes. Plasmid solutions were removed from -20 °C and equilibrated on ice as well. 200 ng plasmid were gently added to the cells and mixed by stirring with a pipette tip. The mixture was incubated on ice for 30 minutes. A heat shock was performed by placing the mixture in a water bath at 42 °C for 45 seconds, followed by recovery on ice for 2 minutes. Subsequently, the cells were suspended in 0.9 mL of SOB media and incubated at 37 °C for 1 hour. After incubation, the cells were centrifuged at 1000 g, and 0.9 mL of supernatant was removed. The cell pellet was then resuspended in the remaining solution and streaked on LB-agar plates supplemented with 150 µg/mL ampicillin using glass beads. Streaked agar plates were incubated at 37 °C overnight.

Precultures

Precultures of single colonies picked from previously prepare LB-agar plates of transformed cells were incubated overnight in 5 mL sterile LB-Miller medium supplemented with 150 µg/mL sodium ampicillin at 37°C and shaking at 180 rpm. The *E. coli* cells for each protein are listed here:
Enc$^{(neg)}$: *E. Coli* C43 (DE3) (NEB)
Ftn$^{(pos)}$: *E. Coli* BL21-Gold (DE3) (Agilent)

Production Cultures

For Enc(neg), 4 mL of the preculture are added to 400 mL terrific broth (TB) containing ampicillin (150 µg/mL). The cells were grown at 37 °C and 180 rpm to an OD_{600} of 0.7. Protein overexpression was induced by isopropyl-β-D-1-thiogalactopyranoside (IPTG) at a final concentration of 0.5 mM. After 24 h expression at 37 °C, cells were harvested via centrifugation at 4000 g. Pellets were stored at -20 °C until further use.

For Ftn(pos), 400 mL LB medium were supplemented with 4 mL preculture and ampicillin (150 µg/mL) and incubated at 37 °C and 180 rpm. Cell cultures were grown to and OD_{600} of 0.2. Protein overexpression was induced with IPTG (0.25 mM). After 5 h incubation at 37 °C, cells were harvested by centrifugation at 4000 g for 20 min. Cell pellets were stored at -20°C until further use.

Protein Purification

Cells from Enc(neg) were resuspended in 20 mL of buffer (50 mM Tris pH 9.0, 0.3 M NaCl), supplemented with 30 mg of RNase A and 15 mg of DNase I. Cell lysis was achieved by subjecting the suspension to 15 rounds of sonication at 60% amplitude for 1 minute each, on ice, with 1-minute breaks between each sonication step, using a Vibra-Cell VCX-130 Ultrasonic Processor (Sonics). The lysed suspension was then centrifuged at 14000 g for 20 minutes, and the pelleted cell debris was discarded. The resulting supernatant was supplemented with $MgCl_2$ (2.5 mM) and $CaCl_2$ (0.5 mM) before incubating for at least 4 hours at 37 °C. Subsequently, the solution was cleared by centrifugation at 14000 g for 15 minutes. Any residual *E. Coli* proteins were denatured by heating the solution for 15 minutes at 65 °C in a water bath and removed by subsequent centrifugation at 14000 g for 15 minutes. The proteins remaining in the solution were precipitated with ammonium sulfate to a final concentration of 70% of its saturation. After centrifugation at 14000 g for 20 minutes, the resulting protein pellet was redissolved in 10 mL of buffer (50 mM Tris pH 9.0), and a second ammonium sulfate precipitation step was carried out. The resulting protein pellet was then dissolved in 50 mL of buffer (50 mM Tris pH 9.0) and subjected to ion-exchange chromatography using a 5 mL HiTrap Q HP anion exchange column (Cytiva). A linear gradient from 0 M to 1 M NaCl was used. Enc(neg) fractions were collected and concentrated to a final volume of 1.0 mL using a centrifugal filter unit. Further purification was performed on a Superose 6 Increase 10/300 GL gel filtration column (Cytiva) in SEC buffer (20 mM Tris pH 7.5, 0.3 M NaCl). For crystallization, a total of three runs were performed on a sample to achieve high purity.

For purification of Ftn$^{(pos)}$, a cell pellet was resuspended in 20 mL of buffer (50 mM Tris pH 7.5, 1.0 M NaCl). The suspension was sonicated six times for 1 minute each (at 60% amplitude) with 1-minute breaks in between, using a Vibra-Cell VCX-130 Ultrasonic Processor (Sonics). Next, the lysed suspension was centrifuged at 14000 g for 15 minutes. The resulting supernatant was incubated with 25 mg of RNase A at 37 °C for 3.5 hours for digestion. After digestion, the solution was centrifuged again at 14000 g for 15 minutes. Subsequently, the supernatant was heated at 65 °C in a water bath for 10 minutes and centrifuged at 14000 g for another 15 minutes. Saturated ammonium sulfate was added to the solution until 70% of its saturation was reached, and the solution was centrifuged again at 14000 g for 20 minutes. The resulting pellet was redissolved in 10 mL of buffer (50 mM MES pH 6.0, 0.5 M NaCl) and then precipitated again with saturated ammonium sulfate. Finally, the pellet was resuspended in 50 mL of buffer (50 mM MES pH 6.0, 0.5 M NaCl). For ion-exchange chromatography (IEC), the sample was purified on a 5 mL HiTrap SP HP cation exchange column (Cytiva) with a linear gradient from 0.5 M to 1.5 M NaCl using 50 mM MES pH 6.0 buffers. The Ftn$^{(pos)}$ containing fractions were collected and concentrated to a final volume of 2 mL. The sample was further purified via size-exclusion chromatography (SEC) on a Hiload 16/600 Superdex 200 PG gel filtration column (Cytiva) using SEC buffer (50 mM Tris pH 7.5, 1.0 M NaCl). Two additional SEC runs were performed for protein crystallization.

Fast protein liquid chromatography – general purification procedures

All protein samples were purified with the respective columns given in the previous section. Protein samples were purified using ion-exchange chromatography, followed by size-exclusion chromatography. Whereas the first method selectively binds charged species, the second method separates samples based on size. This way, highly pure samples were achieved. Generally, for IEC and SEC, only the desired fractions of the main peak (see figure captions for details) were selected. By repeating the SEC purification procedure (two or three times, respectively), monodisperse samples (indicative by sharp, symmetric peaks in the chromatogram) were prepared for further assembly.

Fast protein liquid chromatography – calibration and analysis

A batch crystal sample was centrifuged for 2 minutes at 6000 g. The resulting supernatant (200 µL) was transferred into a new tube and again centrifuged for 2 minutes at 6000 g. The tube containing the batch crystals was stored for other experiments. Next, 150 µL of the

supernatant was taken and diluted with 850 µL Ftn$^{(pos)}$ SEC buffer (50 mM Tris pH 7.5, 1 M NaCl). The combined volume of 1 mL was injected into FPLC for SEC run, using a Superose 6 Increase 10/300 GL gel filtration column (Cytiva) with Ftn$^{(pos)}$ SEC buffer as the elution buffer. In this approach, 1 mL of protein sample with distinct amount of protein was analyzed via SEC for calibration and prepared as batch samples. For each point, the measurements were repeated three times. For calibration measurement points, a different Ftn$^{(pos)}$ variant but with similar structural and physical properties was used for FPLC runs. The evaluation tools of the Cytiva software Unicorn were used to determine the peak area of the chromatograms.

SDS gel electrophoresis – calibration and analysis

The tube containing the batch crystals from experiments described above was used for this analysis. 100 µL of Ftn$^{(pos)}$ SEC buffer was added to a tube containing batch crystals, followed by vortexing and centrifugation. The resulting volume of 100 µL constituted a sample (batch). 10 µL of the sample was mixed with 10 µL SDS loading buffer (50 mM Tris pH 6.8, 10 % (v/v) glycerol, 0.1 % (w/v) bromophenol blue, 2 % (w/v) SDS and 250 mM DTT). Afterwards, the sample was heated for 10 minutes at 95 °C. In cases where bands on the gel appeared too concentrated, 2 µL of the sample were mixed with 18 µL Ftn$^{(pos)}$ SEC buffer, and then 20 µL SDS loading buffer was added. For calibration, distinct amounts of proteins were prepared for SDS gel analysis and prepared as batch crystal samples.

For SDS-PAGE electrophoresis, the apparatus was set to run at a constant voltage of 180 V for at least 90 minutes. Precast Tris-Glycine (4 % - 20 %) gels were purchased at Biotrend. Loading included 10 µL of SDS ladder and 10 µL of each sample. In the staining process, the gel underwent fixing using a destaining solution for 10 minutes, with the solution subsequently poured back into its bottle. Staining was then performed using a staining solution for 15 minutes, followed by pouring the solution back into its bottle. The first destaining step involved using a destaining solution for 15 minutes, and the solution was poured back into its bottle. The second destaining step utilized fresh destaining solution for 30 minutes, with the solution then poured back into the used destaining solution bottle. The final destaining step included destaining the gel for 2 hours, after which the destaining solution was discarded.

Dynamic light scattering (DLS)

DLS measurements were performed on a Zetasizer Pro Blue from Malvern Panalytical equipped with a HeNe laser (λ = 632.8 nm) at a backscattering angle of 173°. Measurements were conducted at 25 °C in ZEN0040 disposable polystyrene cuvettes (Malvern Panalytical). Protein samples were stored in the respective SEC protein buffer. The protein concentration was around 1 mg/mL. For all samples, a refractive index of 1.45 was applied. The freshly purified samples were equilibrated for 120 s, three measurements were performed and averaged. For data analysis, ZS XPLORER software (Malvern Panalytical) was used, with reported hydrodynamic diameters ($D_{hyd.}$) representing the volume-weighted peak means for volume-weighted distributions.

Zeta (ζ) potential measurements

Zeta potential measurements were performed on a *Zetasizer Nano S* from *Malvern Instrument* equipped with HeNe laser (633 nm). Measurements were conducted at 25 °C in micro zeta disposable capillary cells (DTS1070, *Malvern Instrument*). Protein samples were stored in the respective storage buffer. The protein concentration was 1 mg/mL. The samples were equilibrated for 120 s and at least three measurements were performed and averaged. For data analysis, the SMOLUCHOWSKI approximation is applied to convert the electrophoretic mobility to the zeta potential.

Small-angle X-ray scattering and diffraction

Protein crystals of empty $Enc^{(neg)}$ and $Ftn^{(pos)}$ cages were stabilized by using Sulfo-SMCC. The crystal batch was prepared as described above (see main part) and centrifuged for 2 min at 0.1 g to pellet the crystals. 100 µL of the supernatant were mixed with 1.4 mg Sulfo-SMCC and supplemented with 300 µL ultrapure water. 200 µL of this Sulfo-SMCC stock solution was added into the batch. The crystals and solution were gently shaken and incubated for 16 h at 20 °C. Afterwards the cross-linked crystals were washed. First, the tube is centrifuged for 2 min at 1000 g to remove the supernatant. To wash the crystals and remove residual cross-linker 300 µL of ultrapure water were added. After resuspension of the crystals, the process is repeated four times. Then the crystals were stored at 20 °C until further usage.

For SAXS experiments crystals were transferred into Kapton capillary (Goodfellow Cambridge Ltd., England). The capillary was mounted onto a capillary holder. No rotation was performed, due to high amount of sample. SAXS data were collected with a Dectris Eiger2 X 9M at Beamline P62 (DESY, Germany) at 12 keV (1.0332 Å). The measurement was carried out at room temperature. Sample-to-detector distance was 6.24 m. A silver behenate standard sample was used for the calibration of the length of the scattering vector *q*. Azimuthal averaging of the 2D scattering data results in 1D SAXS data (Figure 1). Data processing was carried out by using PyFAI-calib2, a graphical user interface based on PyFAI.[3]

The unit cell parameter *a* for the data set was determined using the scattering vector *q* for the (100) reflection. The relation between the scattering angle θ and the scattering vector q ($q = 4\pi \sin\theta/\lambda$) were combined with the quadratic Bragg equation for cubic crystal systems to obtain equation 1.1:

$$q_{\mathrm{hkl}} = \frac{2\pi}{a}\sqrt{h^2 + k^2 + l^2} \quad (1.1)$$

The cell parameter *a* was determined by using the (100) reflections:

$$a = \frac{2\pi}{q_{100}}\sqrt{h^2 + k^2 + l^2} \quad (1.2)$$

The crystals form a primitive cubic lattice (possible space groups e.g. *P*23 or *P*432). We have determined the unit cells parameters for binary crystals composed of $\mathrm{Enc}^{(\mathrm{neg})}$ and $\mathrm{Ftn}^{(\mathrm{pos})}$ where both cages were empty with dimension *a* = 244.6 Å, measured at room temperature. This is very close to the unit cell determined with low-resolution single-crystal data, measured at 100 K. With this unit cell parameter and equation 1.1., the expected *q* values were calculated (q_{theo}) and plotted versus the measured *q* values (Figure 1).

For the crystals with cages loaded with nanoparticles, the data were processed and scaled with XDS[4] as for the empty cages. The indexed reflections were averaged to yield a peak for each reflection and plotted in Figure 5 (black lines). The determined unit cell parameters were used to simulate diffraction patterns (Figure 5) with Powder Cell[5] (Federal Institute for Material Research and Testing, Germany). For the simulation, a tenth of the lattice parameters were used and the peak position was corrected during the conversion from scattering angle to scattering vector. Simulated diffraction patterns were plotted together with experimental data in Origin (Origin Lab Corporation).

Electrospray ionization mass spectrometry

Protein samples were rebuffered to ultrapure water using a centrifugal filter (Amicon® Ultra-0.5 MWCO 30 kDa from Merck). The sample was filled with ultrapure water to its maximum

volume and concentrated until the dead volume was reached. This process was repeated five times to achieve a protein concentration of approximately 2 mg/mL. Molecular mass of protein was determined using electrospray ionization (ESI) time-of-flight mass spectrometry (Agilent 6224 ESI-TOF). The measurement was conducted by the MS Service at the University of Hamburg, Germany, in positive mode, with a tracked mass range of m/z values between 110 and 3200 at a rate of 1.03 spectra per second. The instrument's source temperature was set to 325 °C, the gas flow at 10 L/min, and the nebulizer pressure at 15 psig. The capillary voltage was set to 4 kV. Data interpretation was carried out with the software MestreNova.

UV-Vis spectroscopy

Absorbance spectra were recorded on a Cary 60 UV-Vis spectrophotometer manufactured by Agilent. Disposable UV microcuvettes (Brand) with 1 cm path length were used. As reference either ultrapure water or respective buffer acted as reference.

Scanning transmission electron microscopy

To image protein crystals in transmission using the STEM detector of the Nova Nano SEM[6] lamellae were prepared by ion beam milling in the SCIOS FIB-SEM using Ga ions at an acceleration voltage of 30 kV. First, a protective Pt layer was applied on the selected region of interest via electron and ion beam induced deposition (EBID and IBID) of a Pt-containing precursor. Then, two wedges were prepared on either side of the lamella still fixed to the crystal on the other two sides and the bottom at an ion current of 3 nA. Subsequently, the lamella was glued to the micromanipulator via IBID. Then, with an undercut, the lamella was loosened from the bottom with the Ga ion beam and further cut from the two sides, such that it was only attached to the micromanipulator and freely moving. Then, the lamellar was lifted out, transferred to a STEM sample holder, and glued for the final thinning with the Ga ion beam at a current of 0.3 nA down to around 50-150 nm. The final lamella as fixed to the STEM sample holder was transferred to the SEM, where it was analyzed at different magnifications and acceleration voltages in between 5-20 kV in several imaging modes including SE imaging with the EDT detector, the through-lens detector in SE and in backscattered electron (BE) imaging mode, as well with a concentric lens-mounted BE detector. Finally, the lamellar was imaged in transmission geometry with an automatically retractable STEM detector permitting to analyze the lamella in varies imaging modes including bright and dark field, and high-angle annular dark-field (HAADF).

Atomic force microscopy

The top surface of the dried protein crystals on the silicon wafer were investigated in air with a Dimension Icon AFM from Bruker.[6] Standard oxide-sharpened tapping mode cantilevers with an Al reflective coating on the backside (RTESPA-300, Bruker, nominal tip radius 8 nm, nominal resonance frequency 300 kHz) were used to map single nanocage crystals pre-selected in the optical microscope. Height, amplitude and phase images were simultaneously recorded for different protein nanocage samples and different crystals at a scanning speed of 1 Hz and an image resolution of 128 × 128 (overview images) 256 × 256 (high resolution images). To consider potential sample tilts from mounting, a second order plane fit was applied.

Supporting Figures

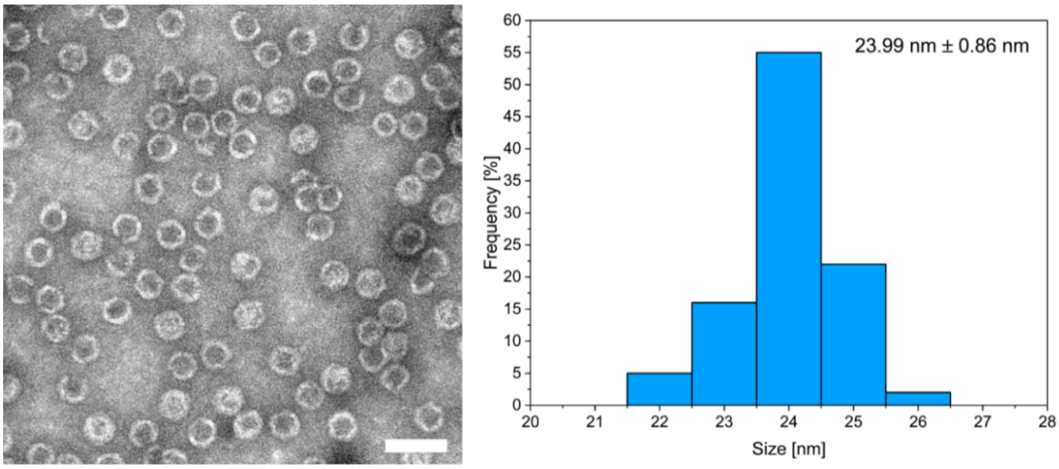

Figure S1: Stained TEM micrograph of Enc$^{(neg)}$ with histogram. Protein cages have a size of roughly 24 nm. Scale bar equals 50 nm.

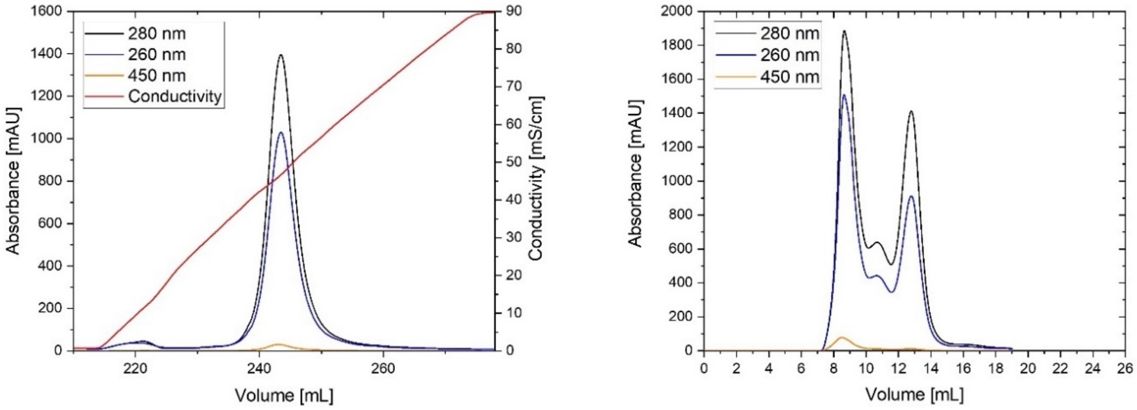

Figure S2: Protein purification of Enc$^{(neg)}$. The left image shows an ion-exchange chromatogram and on the right the consecutive size-exclusion chromatogram. The elution volume of Enc$^{(neg)}$ is between 12 and 13 mL.

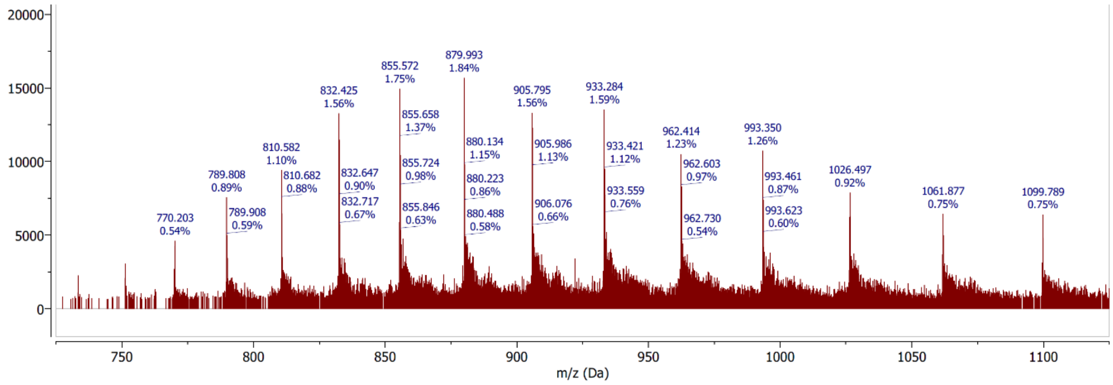

Figure S3: ESI MS spectra to characterize Enc$^{(neg)}$. The determined molecular mass equals to 30764.76 g/mol, which is very close to the expected value of 30765.18 g/mol. Since the encapsulin cage is constructed out of 60 subunits the total molecular mass is 1845910.80 g/mol.

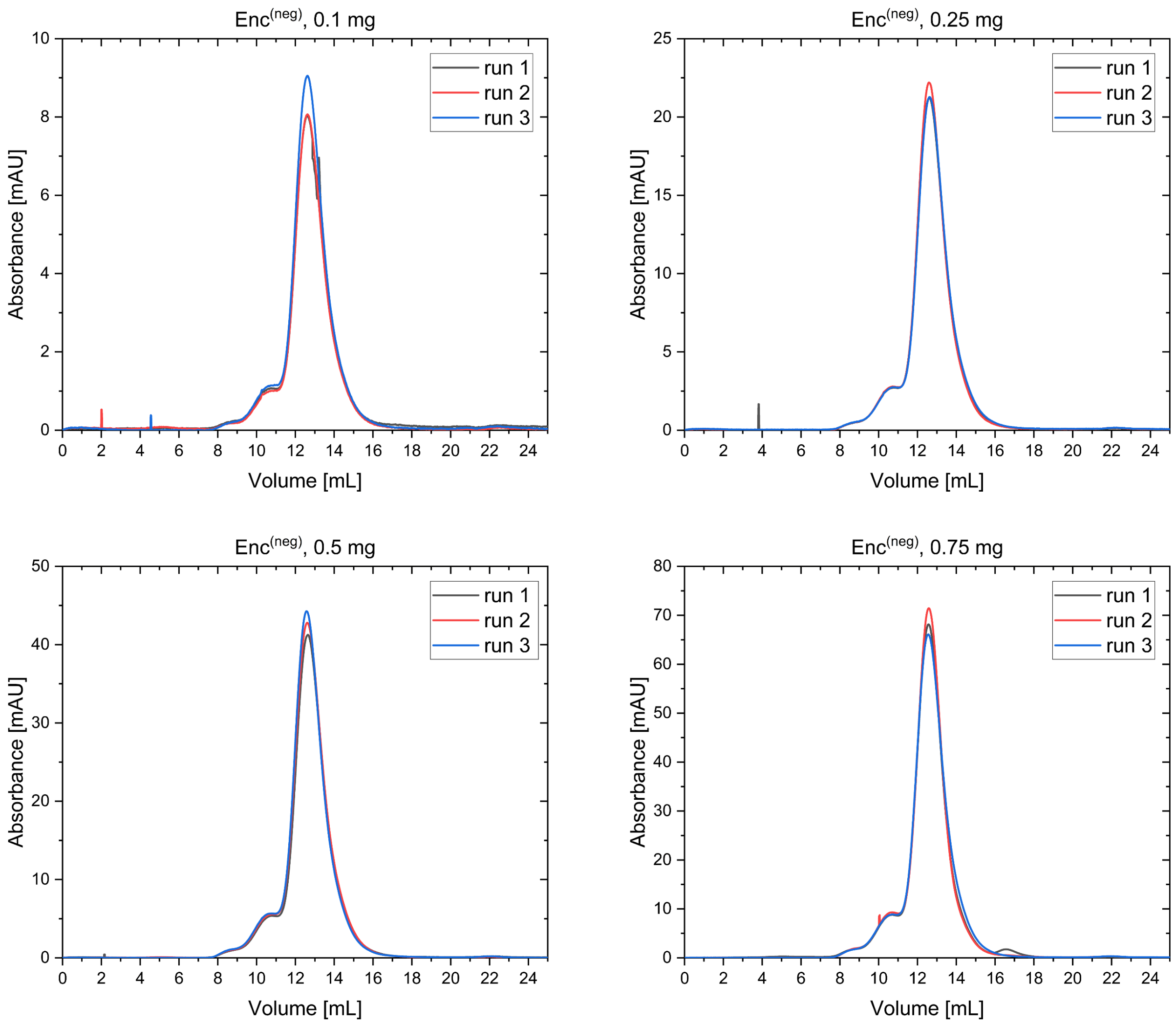

Figure S4: Size-exclusion runs of Enc$^{(neg)}$ for chromatography calibration. For each of the four protein samples, three runs were performed to be averaged later.

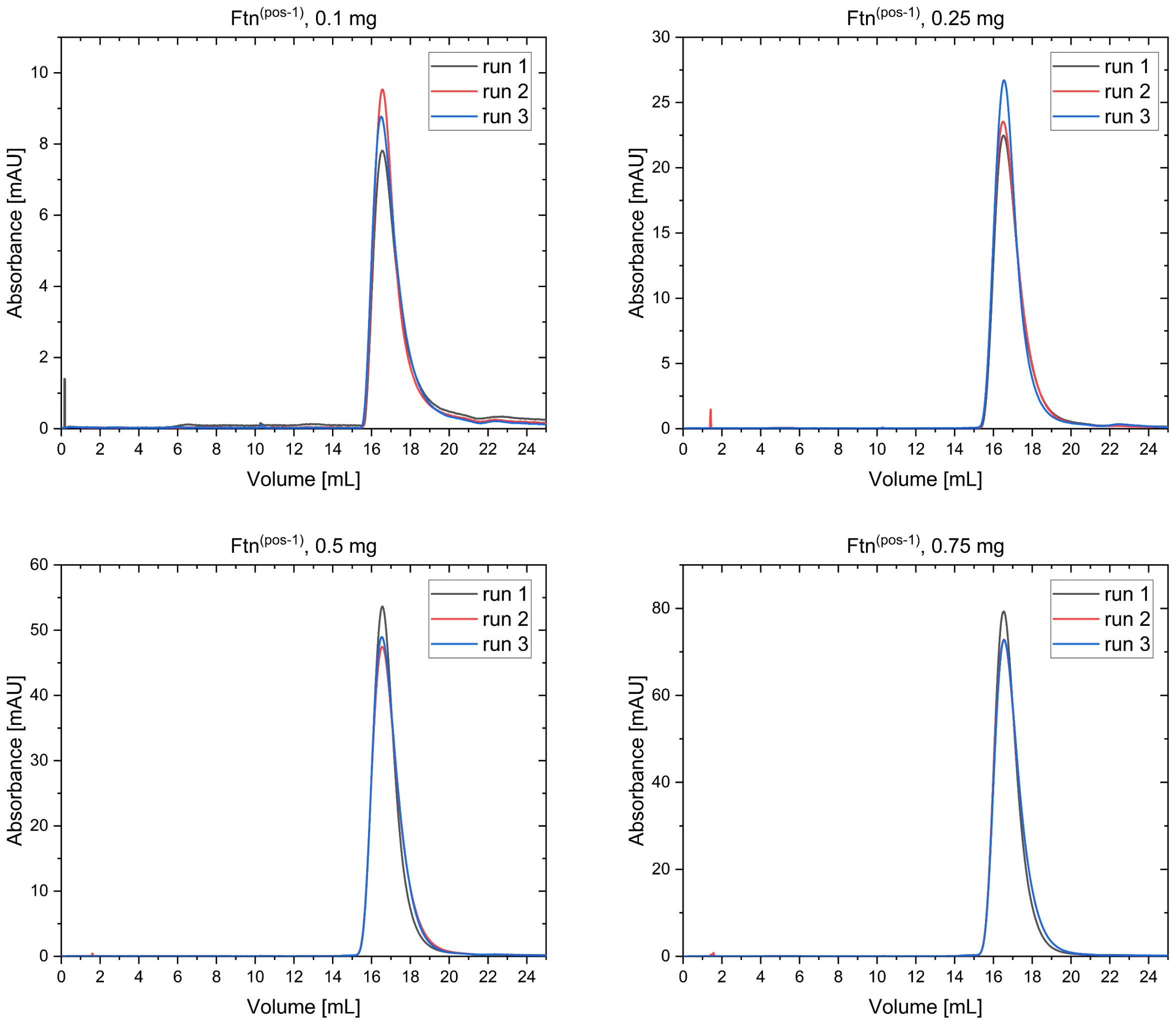

Figure S5: Size-exclusion runs of Ftn$^{(pos)}$ for chromatography calibration. For each of the four protein samples three runs were performed to be averaged later.

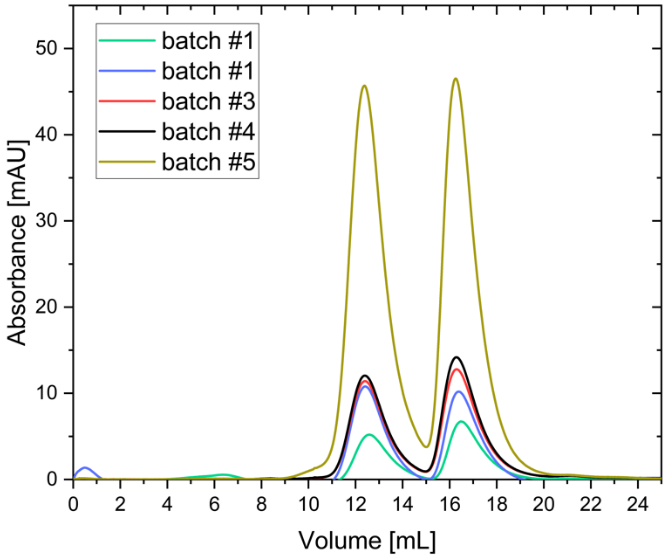

Figure S6: Size-exclusion runs of all five batch samples. For each of the five protein samples the peak area was determined to determine the protein content.

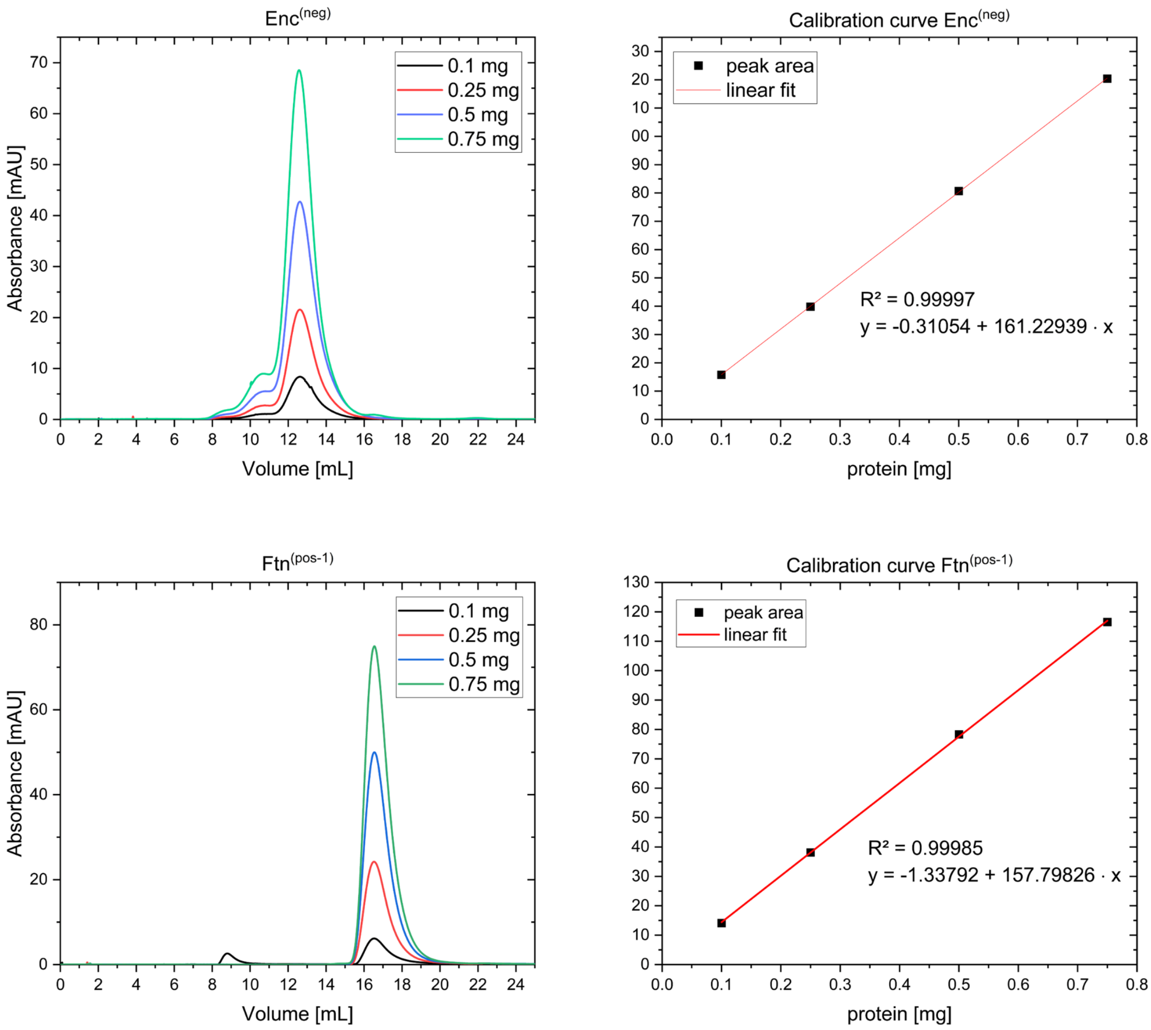

Figure S7: Averaged SEC runs with corresponding calibration curves. For each protein, all three runs per protein mass were averaged and plotted (left column). The calibrations curves based on these datapoints can be used to determine the amount of protein in batch crystallization samples.

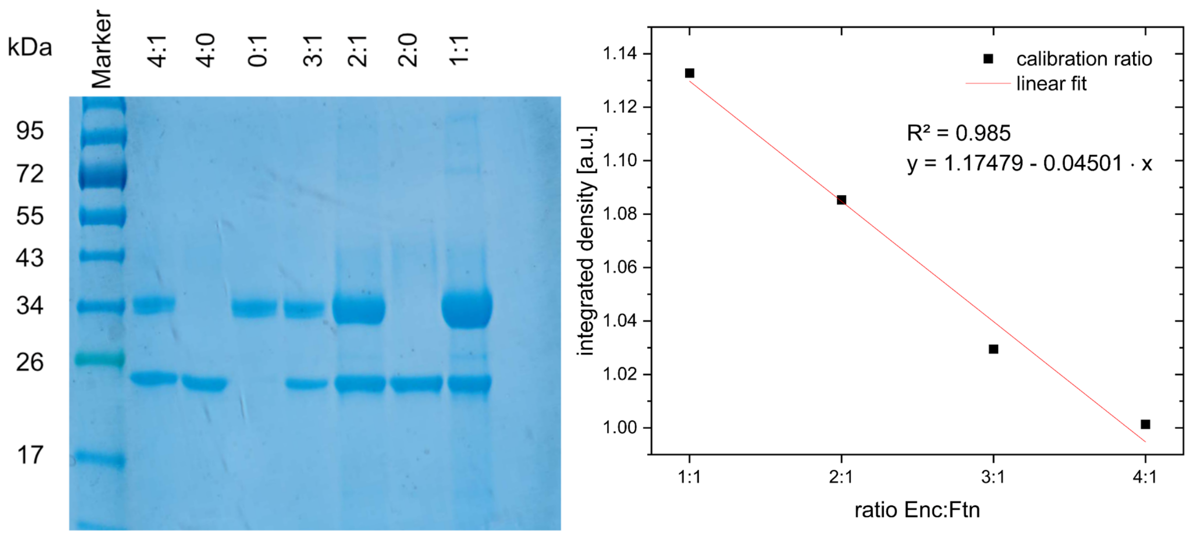

Figure S8: SDS gel and calibration curve. Mixtures of different ratios of Enc$^{(neg)}$ and Ftn$^{(pos)}$ were analyzed via SDS gel. The software ImageJ was used to calculate the integrated density of a protein band. Then, ratios between the integrated density of an encapsulin and ferritin band are calculated. Additionally, samples with only one protein were also given to SDS gel analysis to compare if the integrated density significantly differs if the opposite protein is missing. The calibration will then be needed to analyze batch crystallization samples.

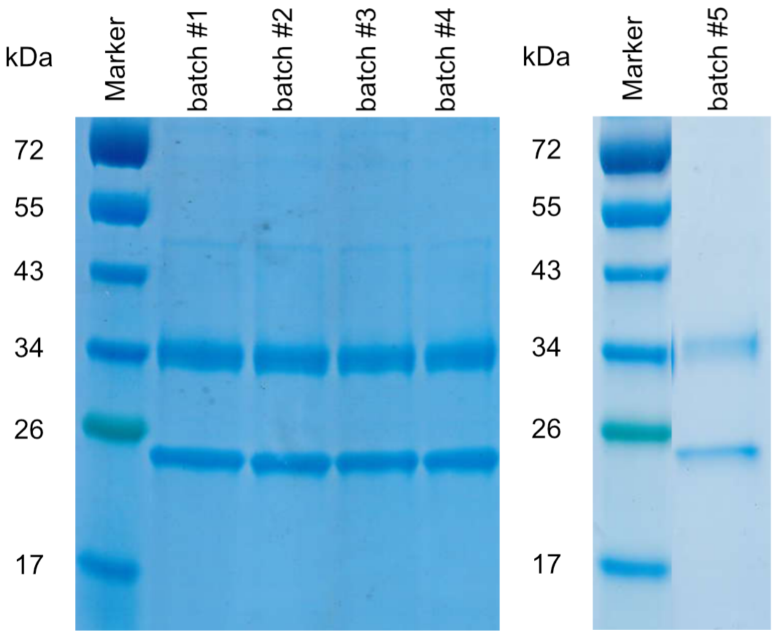

Figure S9: SDS gels of batch crystallization samples. The solution of dissolved protein crystals was analyzed via SDS gel. As previously described, the integrated density will be determined and used to determine the protein amount from the batch samples.

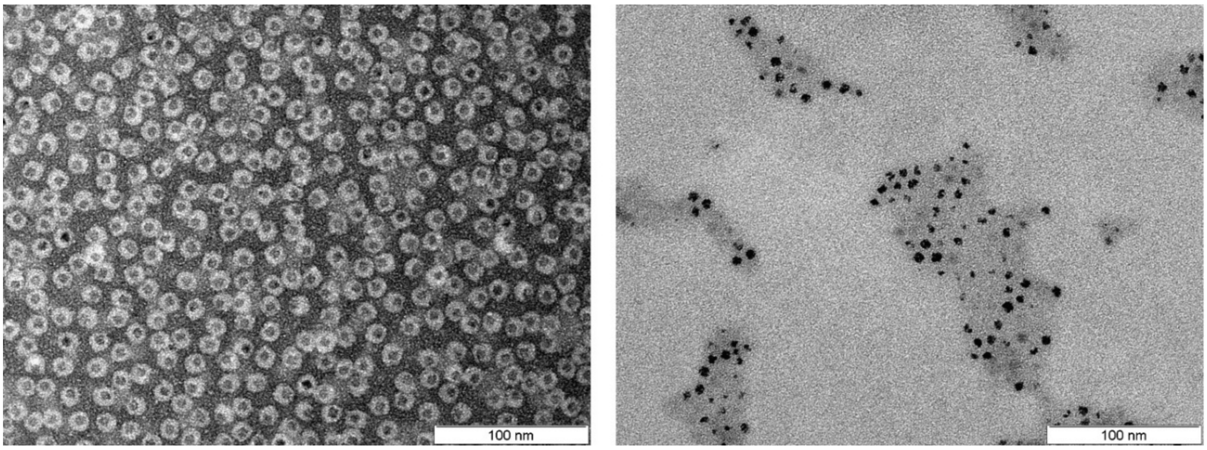

Figure S10: TEM micrograph of CeFtn$^{(pos)}$. Within the stained TEM image of CeFtn$^{(pos)}$ (left) the protein cages and CeO2 NPs are visible, while in the unstained TEM image (right) only the NPs are visible.

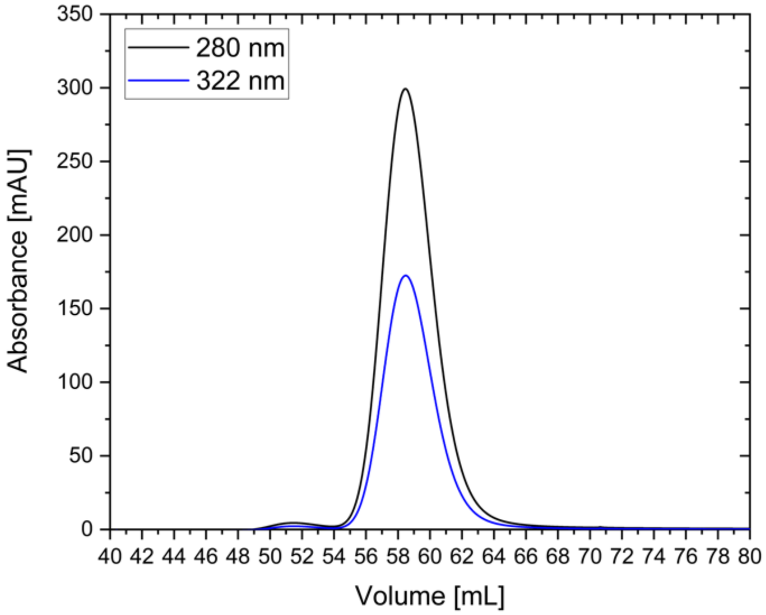

Figure S11: FPLC SEC of cerium oxide nanoparticle loaded Ftn$^{(pos)}$. The NP-loaded ferritin sample elutes around 59 mL. Absorbance at 322 nm is tracked since this wavelength is characteristic for CeO2 NPs.

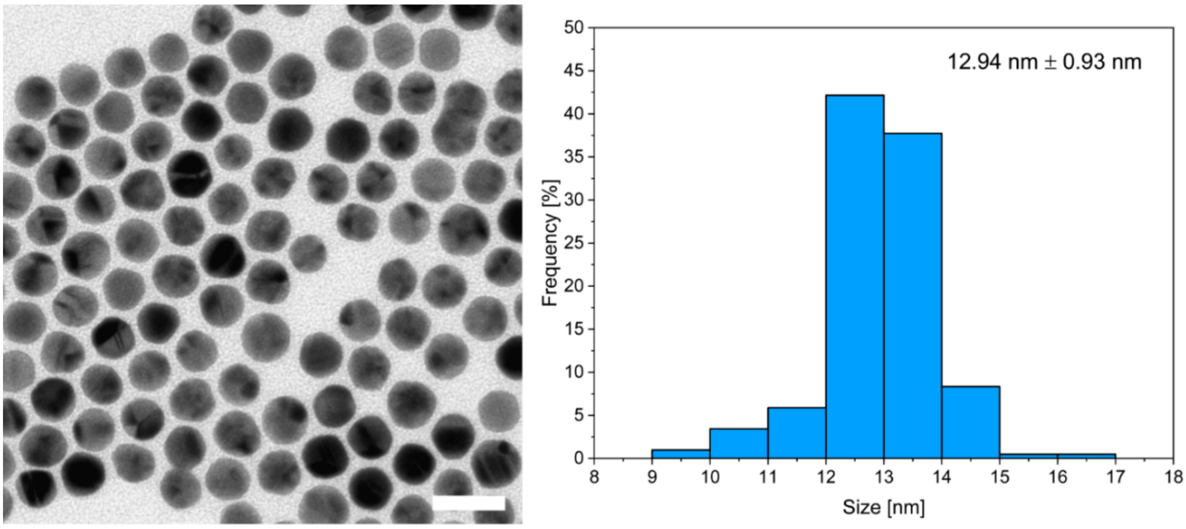

Figure S12: TEM image of synthesized gold nanoparticles with histogram. The synthesized gold nanoparticles have a size of around 13 nm. The scale bar equals 20 nm.

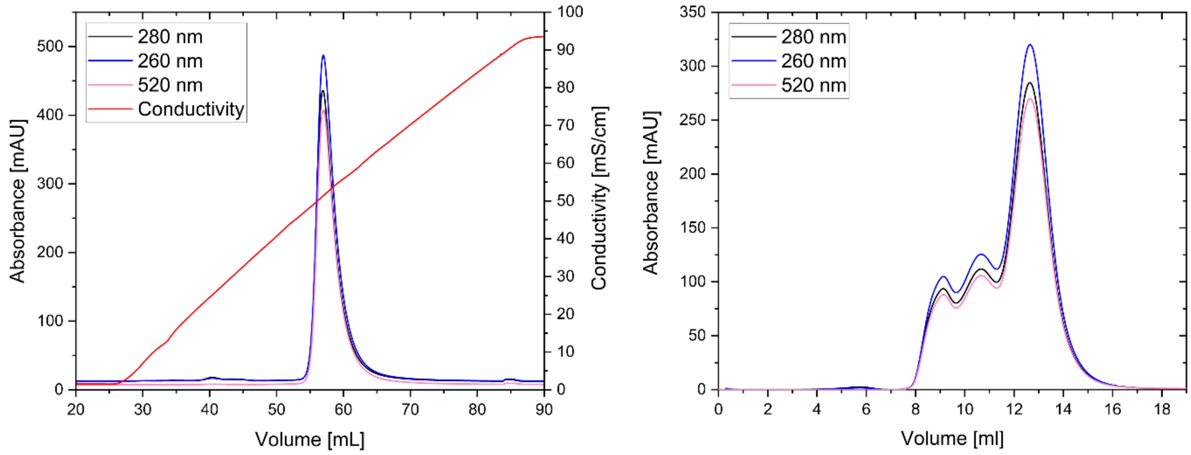

Figure S13: Purification of gold nanoparticle loaded Enc$^{(neg)}$. Left panel: The protein sample is first purified via ion-exchange chromatography to separate protein from free nanoparticles (positively charged particles do not bind to this column), Right panel: Consequently protein aggregates are removed from monomeric protein cage sample through SEC (elution volume between 12 and 13 mL). Absorbance at 520 nm is tracked due to the plasmonic gold nanoparticle absorption.

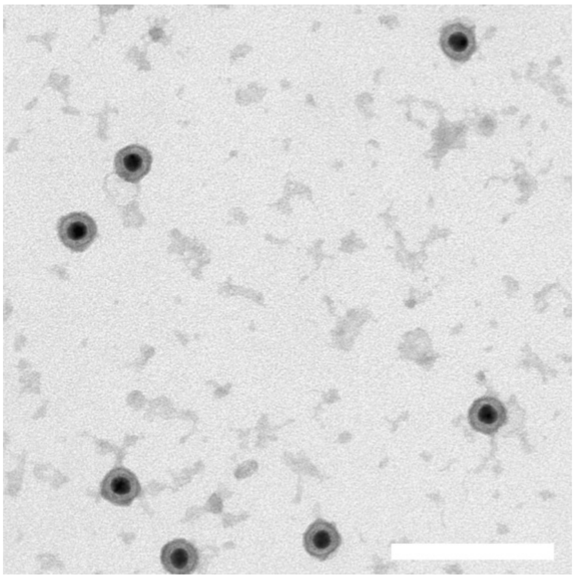

Figure S14: TEM micrograph of AuNP-loaded Enc$^{(neg)}$. Within the stained TEM image of AuEnc$^{(neg)}$ protein cages and NPs are visible. Scale bar is100 nm.

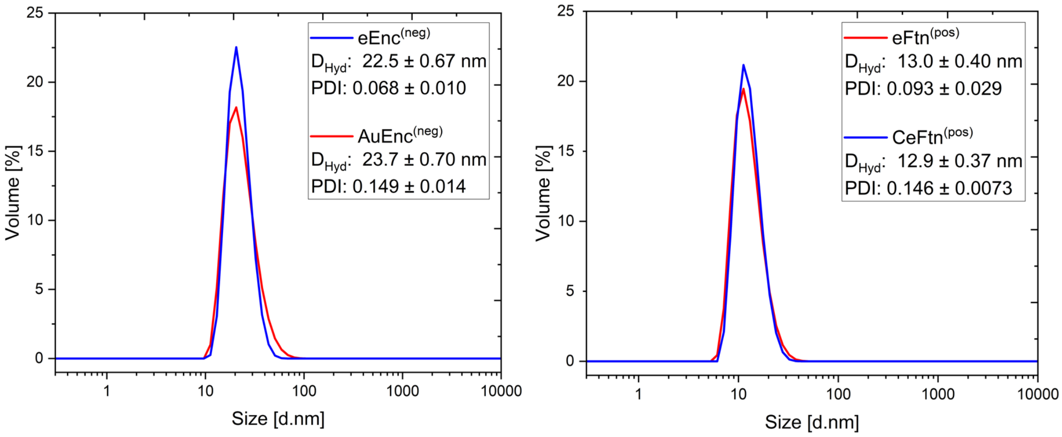

Figure S15: Dynamic light scattering of the building blocks. Left: Data for empty and AuNP-loaded Enc$^{(neg)}$. Right panel: Data for empty and CeO_2-loaded Ftn$^{(pos)}$. The hydrodynamic diameter and the polydispersity index (PDI) are given for each sample.

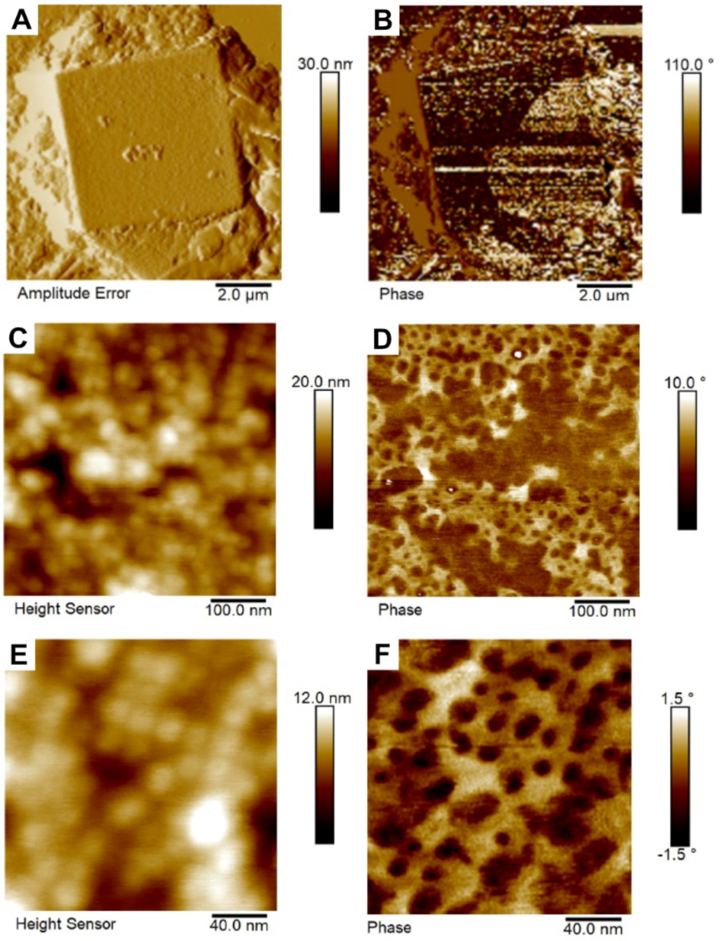

Figure S16: AFM images of hetero binary AuEnc$^{(neg)}$/eFtn$^{(pos)}$ crystal. Within the different images, some contamination on the crystal surface can be observed, but still ordered spherical objects of roughly 24 nm are found (either bright spots in height sensor or dark spots in phases). **A, B**: Overview amplitude and phase images showing the full selected crystal. **C, D**: Height and phase images of intermediate magnification, highlighting regions with ordered patches on the top surface of the crystal next two regions of less or no ordering. **E, F**: High resolution height and phase images of regions with presumably single nanocages exhibiting local order.

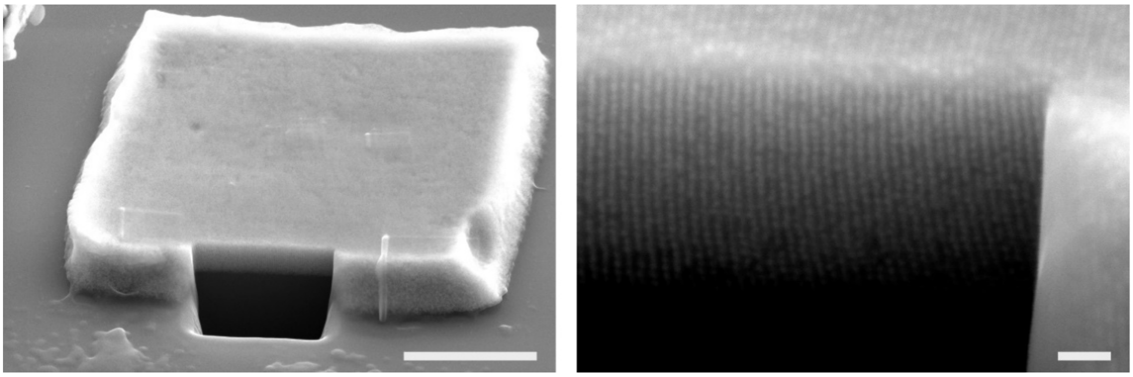

Figure S17: SEM image of a hetero binary AuEnc$^{(neg)}$/eFtn$^{(pos)}$ crystal with a cut cross-section prepared by ion milling. Inside the crystal the lattice could be visualized. Scale bars: 2 µm and 100 nm.

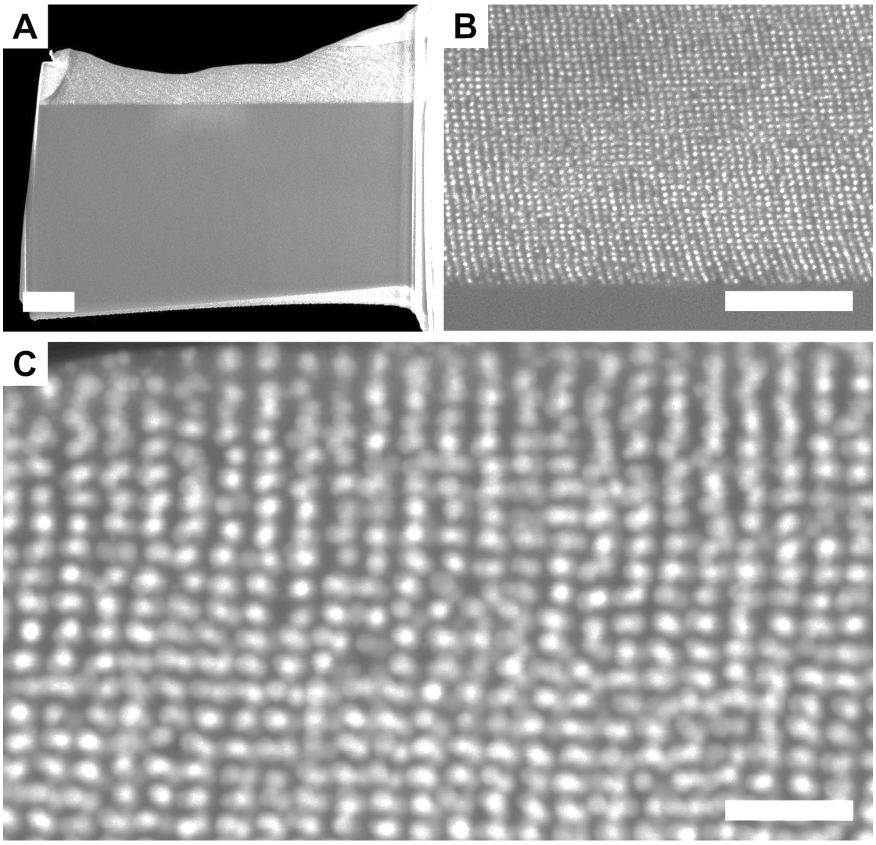

Figure S18: STEM image of hetero binary AuEnc$^{(neg)}$/eFtn$^{(pos)}$ crystal. The protein crystal was milled via FIB and characterized via STEM to visualize the AuEnc$^{(neg)}$ within the lattice. **A**: Overview through-lens secondary electron image of the lamella with the Pt protective coating on top, the protein crystal layer in the center and the silicon crystal support on the bottom. **B**: Further zoom into the crystal reveals the protein lattice. **C**: High-resolution HAADF transmission image taken in the central part of the protein crystal, showing the ordered arrangement of the filled nanocages. Image scale bars: 1 µm, 300 nm and 100 nm for **A**, **B** and **C** respectively.

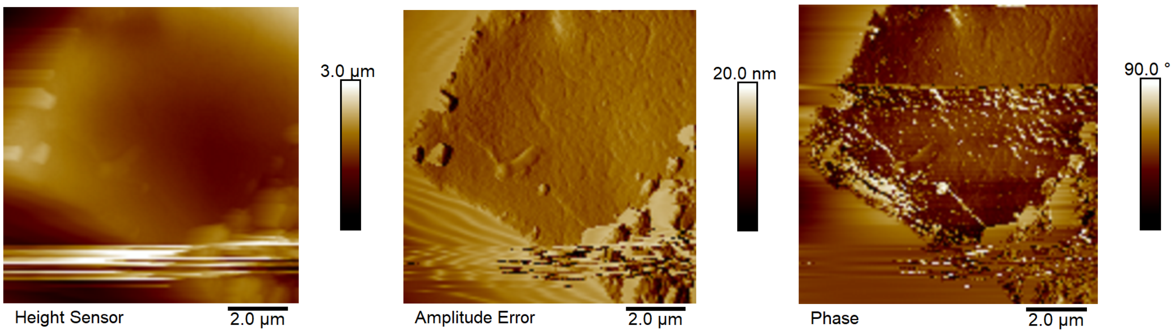

Figure S19: AFM images of hetero binary eEnc$^{(neg)}$/CeFtn$^{(pos)}$ crystal. The surface roughness of these crystals did not enable any visualization of the CeFtn$^{(pos)}$ cages.

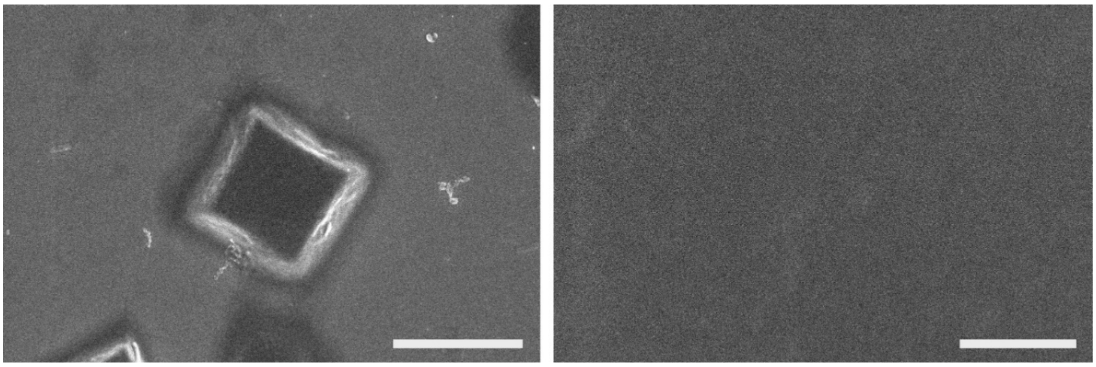

Figure S20: SEM images of hetero binary eEnc$^{(neg)}$/CeFtn$^{(pos)}$ crystal. No visualization of the CeFtn$^{(pos)}$ cages on the crystal surface. Scale bars: 10 µm and 300 nm.

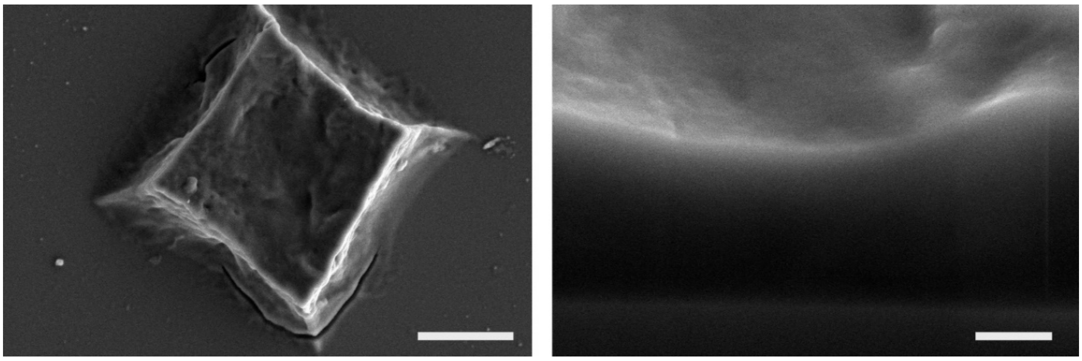

Figure S21: SEM image of hetero binary eEnc$^{(neg)}$/CeFtn$^{(pos)}$ crystal before and after milling. Before (left image) and even after milling via FIB (right image) no CeFtn$^{(pos)}$ could be visualized. Scale bars: 5 µm and 1 µm.

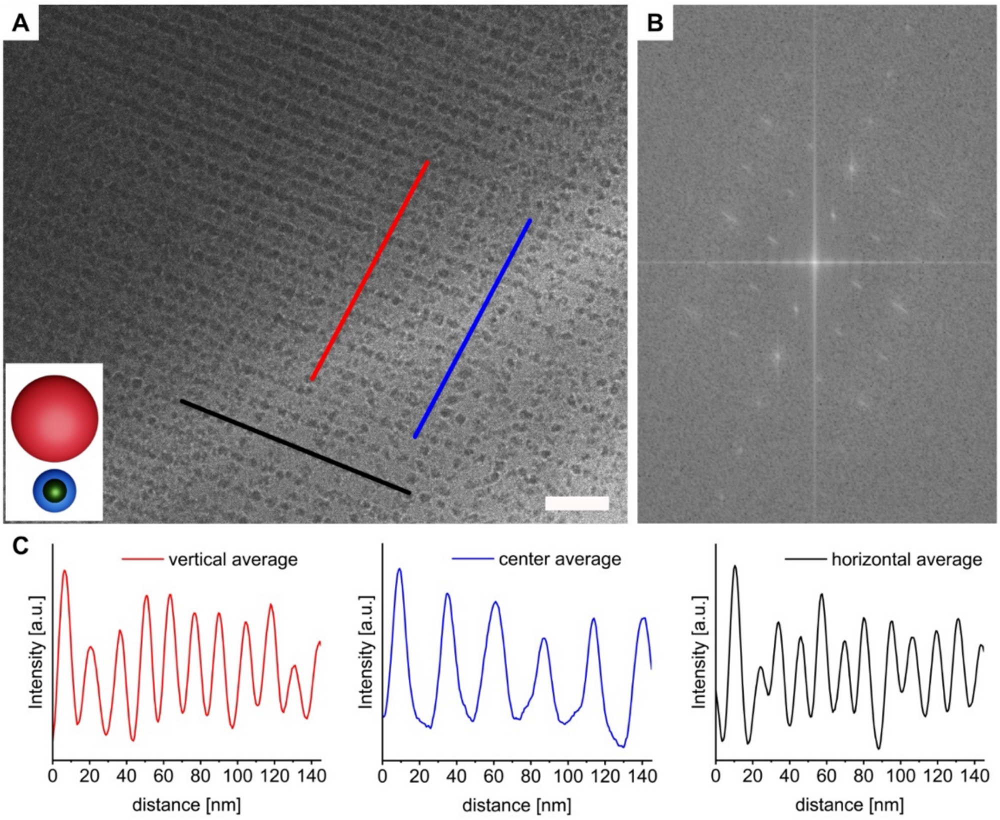

Figure S22. In situ structural characterization of empty Enc$^{(neg)}$ and CeO_2 NP-loaded Ftn$^{(pos)}$ by cryo transmission electron microscopy (cryo-TEM) at high magnification. A: Cryo-TEM micrograph of an exemplary lamella prepared by cryo-FIB from a hetero binary crystal composed of eEnc$^{(neg)}$ and CeFtn$^{(pos)}$. Scale bar is 50 nm. B: The corresponding fast Fourier transformation is depicted. A cubic arrangement can be derived from the observed spots. C: Measuring the distances of objects indicates the arrangement of CeFtn$^{(pos)}$ within the unit cell, while the empty eEnc$^{(neg)}$ is not visible.

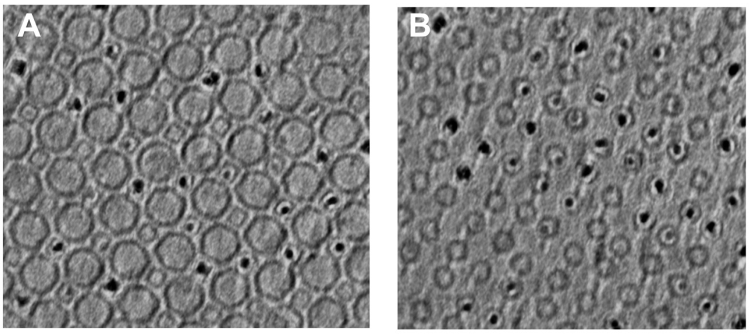

Figure S23: Electron cryo-tomogram of a lamella from an eEnc$^{(neg)}$/CeFtn$^{(pos)}$ crystal. Similar images of the tomogram are shown as in Figure 4A and B, but here without the model (no spheres).

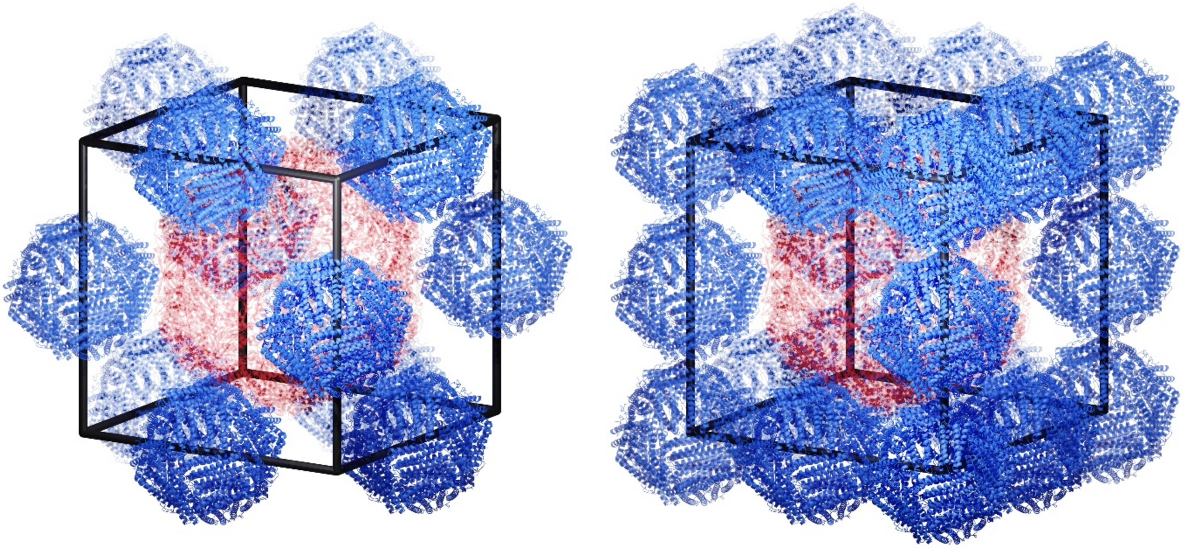

Figure S24: Comparison of an AB_3 and AB_4 unit cell model. Unit cells of possible AB_3 (left) and AB_4 (right) structures are shown. In the AB_3 unit cell, one encapsulin and three ferritins are located. The AB_4 unit cell features four ferritins and one encapsulin.

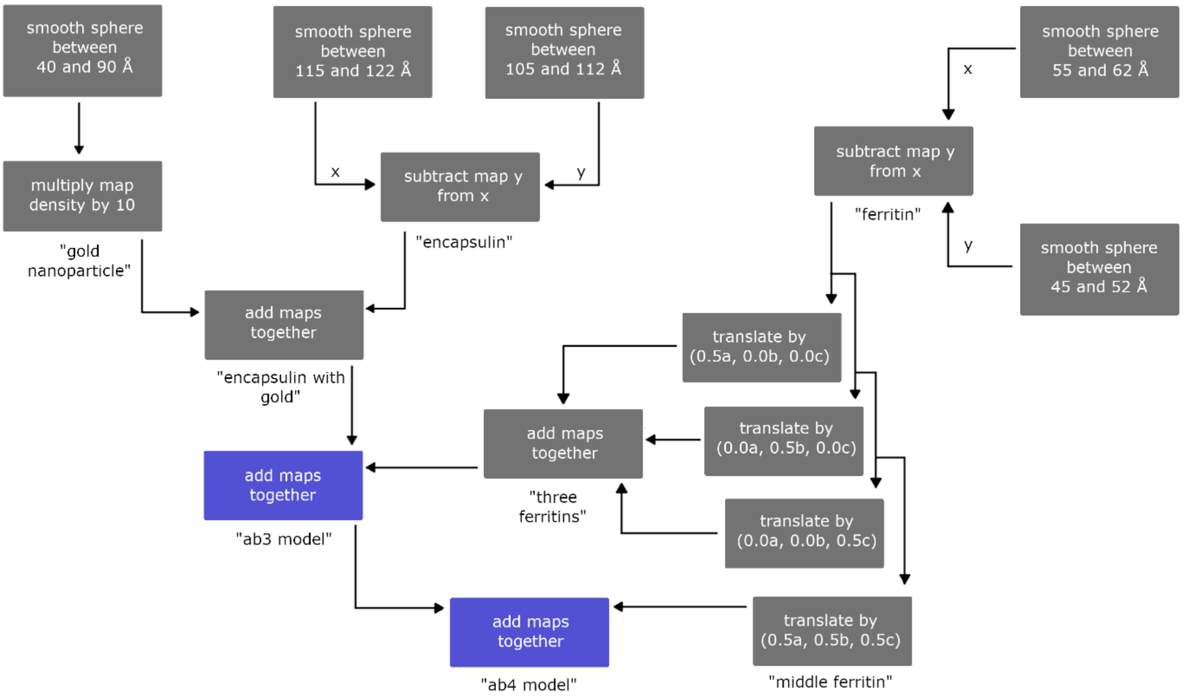

Figure S25: Schematic overview of structure modelling.

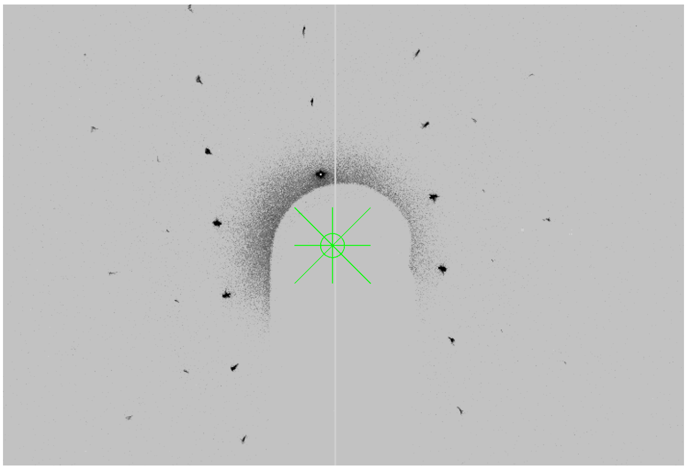

Figure S26: Representative X-ray diffraction image for the AuEnc$^{(neg)}$/eFtn$^{(pos)}$ crystal.

Supporting Tables

Table S1: Sequence of primer used for the mutation of *T. maritima* wild type encapsulin.

Mutation	Forward primer
W90E	CGTTCACACTCGACTTGGAGGAGTTGGACAATCTCG

Table S2: Protein sequences for protein variants.

Protein variant	Protein sequence
Enc$^{(neg)}$	MVNMEFLKRSFAPLTEKQWQEIDNRAREIFKTQLYGRKFVDVEGPYGWEYAAHPL GEVEVLSDENEVVKWGLRKSLPLIELRATFTLDLEELDNLERGKPNVDLSSLEETVR KVAEFEDEVIFRGCEKSGVKGLLSFEERKIECGSTPKDLLEAIVRALSIFSKDGIEGP YTLVINTDRWINFLKEEAGHYPLEKRVEECLRGGKIITTPRIEDALVVSERGGDFKLIL GQDLSIGYEDREKDAVRLFITETFTFQVVNPEALILLKF
Ftn$^{(pos)}$	MTTASTSQVRQNYHQDSEKAINRQIRLELYASYVYLSMSYYFDRDDVALKNFAKYF LHQSHEEREHAEKLMKLQNQRGGRIFLQDIQKPDKDDWESGLRAMEKALKLEKKV NQSLLELHKLATKKNDPHLCDFIETHYLNEQVKAIKELGDHVTNLRKMGAPRSGLA EYLFDKHTLGDSDNES

Table S3: Two-step PCR cycling protocol.

Segment	Cycles	Temperature [°C]	Time	Total time
1	1x	95	30 s	23.5 min
2	3x	95	30 s	
		58	1 min	
		68	6 min	
Hold	-	4	∞	-
3	16x	95	30 s	120 min
		58	1 min	
		68	6 min	
4	-	68	10 min	10 min
Storage	-	4	∞	-

Table S4: Sequence of the cargo-loading peptide.

N-term	CLP sequence	C-term
H	CGGSENTGGDLGIRKL	OH

Table S5: Data points for FPLC calibration for both proteins. The peak area below the protein peaks were determined via Unicorn evaluation tool.

Sample	0.1 mg	0.25 mg	0.5 mg	0.75 mg
Enc$^{(neg)}$	15.043<br>16.995<br>15.280	39.931<br>39.779<br>39.865	77.604<br>82.214<br>82.238	117.663<br>121.005<br>122.559
Ftn$^{(pos)}$	13.464<br>14.414<br>14.484	38.380<br>36.720<br>39.321	78.667<br>77.011<br>79.339	117.499<br>116.902<br>115.175

Table S6: Averaged peak areas of protein samples for calibration.

Sample	0.1 mg	0.25 mg	0.5 mg	0.75 mg
Enc$^{(neg)}$	15.773	39.858	80.685	120.409
Ftn$^{(pos)}$	14.121	38.140	78.339	116.525

Table S7: Evaluation of peak areas from batch samples via peak areas. Based on the peak areas of each protein peak, the amount of protein in the crystal is indirectly calculated.

Sample	Batch 1	Batch 2	Batch 3	Batch 4	Batch 5
Peak area Enc$^{(neg)}$	8.7458	18.1284	21.2684	22.3826	86.2520
Peak area Ftn$^{(pos)}$	11.3260	16.8894	24.0445	26.4950	81.6520
Enc$^{(neg)}$ mg in supernatant	0.0548	0.1437	0.2620	0.1757	0.1414
Ftn$^{(pos)}$ mg in supernatant	0.0803	0.1540	0.1891	0.2352	0.1580
Enc$^{(neg)}$ mol in supernatant	2.9682E-11	7.7850E-11	1.4194E-10	9.5205E-11	7.662E-11

$Ftn^{(pos)}$ mol in supernatant	1.5676E-10	3.0084E-10	3.6931E-10	4.5938E-10	3.087E-10
$Enc^{(neg)}$ mg in crystal	0.5452	0.4563	0.4035	0.4243	0.4586
$Ftn^{(pos)}$ mg in crystal	0.5197	0.4460	0.4582	0.3648	0.4420
$Enc^{(neg)}$ mol in crystal	2.9536E-10	2.4719E-10	2.1859E-10	2.2984E-10	2.4842E-10
$Ftn^{(pos)}$ mol in crystal	1.0152E-09	8.7117E-10	8.9503E-10	7.1263E-10	8.6329E-10
Ratio	3.44	3.52	4.09	3.10	3.48

Table S8: Integrated density values for calibration samples on SDS gel. The values for the integrated density are based on SDS gel bands (Figure S7) and evaluated with calibration curve.

**Sample**	4:1	3:1	2:1	1:1
$Enc^{(neg)}$	1106656	1158389	1023076	967421
$Ftn^{(pos)}$	1108186	1192566	1110397	1095899
ratio	1.001383	1.029504	1.085351	1.132805

Table S9: Integrated density values for five batch crystallization samples. The values for the integrated density are based on SDS gel from Figure S8.

**Sample**	Batch 1	Batch 2	Batch 3	Batch 4	Batch 5
$Enc^{(neg)}$	1702872	1373155	1364186	1391255	1516526
$Ftn^{(pos)}$	1775884	1427568	1403588	1435723	1500758
ratio	1.042876	1.039626	1.028883	1.031963	0.989603
Protein ratio	2.93	3.00	3.24	3.17	4.12

Table S10: Summary of FPLC and SDS data for ratio determination. Determined ratios between Ftn$^{(pos)}$ and Enc$^{(neg)}$ based on either chromatography (FPLC) or electrophoresis (SDS). A mean value between 3 and 4 Ftn to Enc was calculated.

**Protein ratio**	Batch 1	Batch 2	Batch 3	Batch 4	Batch 5	Mean value
FPLC	3.44	3.52	4.09	3.10	3.48	3.53
SDS	2.93	3.00	3.24	3.17	4.12	3.29

Table S11: Unit cell parameters from single crystal SAXD data of nanoparticle-loaded protein crystals

**Crystal**	Unit cell length *a* [Å]	Angles [°]
eEnc$^{(neg)}$ / eFtn$^{(pos)}$	242.57	90
AuEnc$^{(neg)}$ / eFtn$^{(pos)}$	244.48	90
eEnc$^{(neg)}$ / CeFtn$^{(pos)}$	244.00	90

Table S12: Refinement statistics for single crystal SAXS data based on AB$_3$ model.

**NSHELL**	**DMIN**	**DMAX**	**RFACT**	**CORREL**	**<F1>**	**<F2>**	**NREF**
1	244.40	51.31	20.69	0.9465	10.2	10.6	214
2	51.31	40.79	64.02	-0.0151	1.5	2.4	217
3	40.79	35.65	74.39	0.1571	0.8	0.6	231
4	35.65	32.40	34.77	-0.4785	0.8	0.7	218
5	32.40	30.08	59.38	0.1020	0.5	0.4	255
**Overall statistics**			31.90	0.960	2.6	2.8	1134

Table S13: Refinement statistics for single crystal SAXS data based on AB_4 model.

NSHELL	DMIN	DMAX	RFACT	CORREL	<F1>	<F2>	NREF
1	244.40	51.31	21.86	0.9428	10.1	10.6	214
2	51.31	40.79	65.70	0.1035	1.5	2.4	217
3	40.79	35.65	82.68	0.1944	0.8	0.6	231
4	35.65	32.40	43.30	-0.3572	0.9	0.7	218
5	32.40	30.08	61.69	0.0488	0.5	0.4	255
**Overall statistics**			33.90	0.957	2.6	2.8	1134

References

1. Künzle, M.; Mangler, J.; Lach, M.; Beck, T., Peptide-Directed Encapsulation of Inorganic Nanoparticles into Protein Containers. *Nanoscale* **2018,** *10*, 22917-22926.
2. Künzle, M.; Eckert, T.; Beck, T., Binary Protein Crystals for the Assembly of Inorganic Nanoparticle Superlattices. *J. Am. Chem. Soc.* **2016,** *138*, 12731-12734.
3. Ashiotis, G.; Deschildre, A.; Nawaz, Z.; Wright, J. P.; Karkoulis, D.; Picca, F. E.; Kieffer, J., The Fast Azimuthal Integration Python Library: Pyfai. *J. Appl. Crystallogr.* **2015,** *48*, 510-519.
4. Kabsch, W., XDS. *Acta. Crystallogr.* **2010,** *D66*, 125-132.
5. Kraus, W.; Nolze, G., Powder Cell– a Program for the Representation and Manipulation of Crystal Structures and Calculation of the Resulting X-Ray Powder Patterns. *J. Appl. Crystallogr.* **1996,** *29*, 301-303.
6. Stierle, A.; Keller, T. F.; Noei, H.; Vonk, V.; Roehlsberger, R., Desy Nanolab. *JLSRF* **2016,** *2*, A76.
